# Patient-Derived Multiple Myeloma 3D Models for Personalized Medicine—Are We There Yet?

**DOI:** 10.3390/ijms232112888

**Published:** 2022-10-25

**Authors:** Diana Lourenço, Raquel Lopes, Carolina Pestana, Ana C. Queirós, Cristina João, Emilie Arnault Carneiro

**Affiliations:** 1 Myeloma Lymphoma Research Group—Champalimaud Experimental Clinical Research Programme of Champalimaud Foundation, 1400-038 Lisbon, Portugal; 2 Faculty of Medicine, University of Coimbra, 3000-548 Coimbra, Portugal; 3 Faculty of Medicine, University of Lisbon, 1649-028 Lisbon, Portugal; 4 Centre of Statistics and Its Applications, Faculty of Sciences, University of Lisbon, 1749-016 Lisbon, Portugal; 5 Faculty of Medical Sciences, NOVA Medical School, 1169-056 Lisbon, Portugal; 6 Hemato-Oncology Department of Champalimaud Foundation, 1400-038 Lisbon, Portugal

**Keywords:** hematologic cancer, multiple myeloma, bone marrow microenvironment, personalized therapy, 3D models, ex vivo models, primary cell culture

## Abstract

Despite the wide variety of existing therapies, multiple myeloma (MM) remains a disease with dismal prognosis. Choosing the right treatment for each patient remains one of the major challenges. A new approach being explored is the use of ex vivo models for personalized medicine. Two-dimensional culture or animal models often fail to predict clinical outcomes. Three-dimensional ex vivo models using patients’ bone marrow (BM) cells may better reproduce the complexity and heterogeneity of the BM microenvironment. Here, we review the strengths and limitations of currently existing patient-derived ex vivo three-dimensional MM models. We analyze their biochemical and biophysical properties, molecular and cellular characteristics, as well as their potential for drug testing and identification of disease biomarkers. Furthermore, we discuss the remaining challenges and give some insight on how to achieve a more biomimetic and accurate MM BM model. Overall, there is still a need for standardized culture methods and refined readout techniques. Including both myeloma and other cells of the BM microenvironment in a simple and reproducible three-dimensional scaffold is the key to faithfully mapping and examining the relationship between these players in MM. This will allow a patient-personalized profile, providing a powerful tool for clinical and research applications.

## 1. Introduction

Multiple myeloma (MM) is one of the most common hematological malignancies worldwide [1]. It is characterized by bone marrow (BM) infiltration by monoclonal plasma cells and overproduction of monoclonal immunoglobulins, leading to end-organ damage and significant patient morbidity [2,3]. Despite the introduction of several novel drugs, MM remains incurable, with more than 90% of relapsed patients developing drug resistance [4].

Myeloma cells grow within the BM, a hematopoietic organ in which various cell types establish a complex interplay. This involves adhesion molecules and soluble factors that play a critical role in disease progression, making the microenvironment more permissive for immune evasion, proliferation, survival and migration of myeloma cells [5,6,7,8]. Presently, only 5% of the new antitumor molecules end up obtaining clinical approval [9,10], reinforcing the importance of unveiling molecular mechanisms associated with the disease and identifying therapies with less toxicity and meaningful efficacy.

In order to develop the fittest and safest treatment for each individual, a deeper understanding of the underlying disparities between MM patients [11] and of the complex interactions between myeloma cells and their microenvironment is critical. Research performed in conventional two-dimensional (2D) cell culture or in animal models replicates the MM BM milieu sub-optimally, as tissue architecture, tumor surroundings and the crosstalk between cell populations are often not present. This leads to incongruities when compared to clinical outcomes [12,13]. On the other hand, three-dimensional (3D) cell culture models are more capable of providing the proper microenvironment for cell function, growth and differentiation. Therefore, 3D models are emerging as a potential preclinical platform to study the mechanisms of disease and drug testing [14].

MM is mostly driven by cytogenetic abnormalities that involve the translocation of the immunoglobulin heavy-chain gene, in juxtaposition to known oncogenes, such as *CCND1*, *NSD2*, *FGFR3*, *MAF* and *CCND3*, leading to their dysregulation [15]. With disease development, secondary events occur, including somatic mutations and additional copy-number alterations, such as del(17) and del(1p). Specific genetic alterations are already known to be associated with worse prognosis in MM patients, and these are more prone to develop drug resistance [16]. The high inter- and intra-patient heterogeneity, at both genetic and transcriptional levels, makes it difficult to extrapolate very generalized treatment options, and treatments end up not working for every patient [17,18]. Therefore, having ways to examine and evaluate patients in a personalized manner is of outmost importance. Considering patient heterogeneity, the most physiologically relevant 3D culture models should be built on patient-derived samples, as this will allow the study of the disease in each MM patient.

In this review, we first highlight the role of each cell type from the BM microenvironment in MM and how they can be targeted by current therapeutic drugs. We then summarize the existing patient-derived 3D MM models, discussing their strengths and limitations, as well as their potential for exploring molecular mechanisms underlying MM and new therapeutic targets’ discovery. Finally, we provide some understanding of the challenges and solutions for recreating a more comprehensive and reliable MM BM model.

## 2. The Importance of MM Bone Marrow Microenvironment

**Figure 1:**
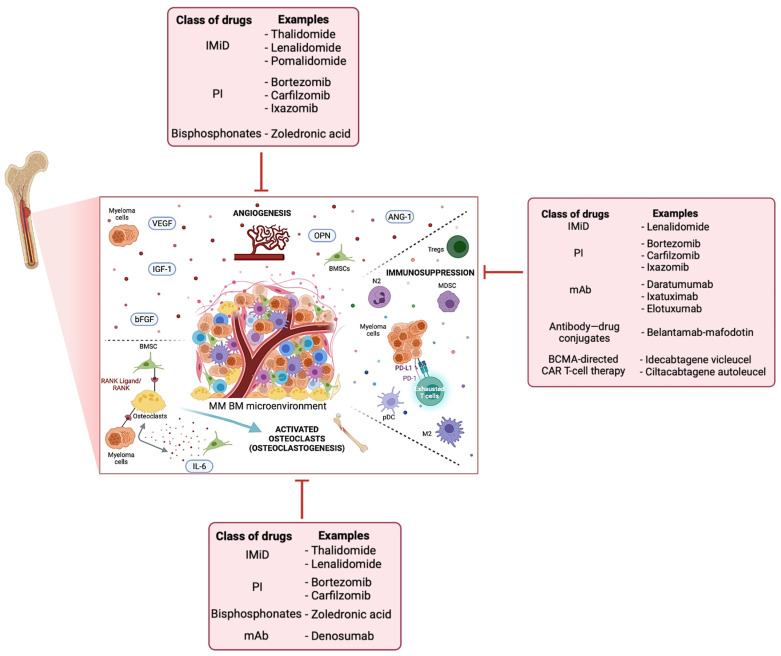
Alterations found in the MM BM niche and therapeutic options to counteract MM. The central box recapitulates the main dysfunctions of the BM microenvironment upon MM establishment, namely angiogenesis, immunosuppression and osteoclastogenesis (separated by dotted lines). The pink boxes summarize the action of different classes of approved drugs used to inhibit dysregulated pathways in the BM microenvironment or to induce a cytotoxic immune response against myeloma cells. Created with BioRender.com. Abbreviations: Ang-1, angiopoietin-1; BCMA, B-cell maturation antigen; bFGF, basic fibroblast growth factor; BMSC, bone marrow stromal cells; CAR, chimeric antigen receptor; IGF-1, type-1 insulin-like growth factor; IL-6, interleukin 6; IMiD, immunomodulators; M2, macrophages type 2; mAb, monoclonal antibodies; MDSC, myeloid-derived suppressor cells; N2, neutrophils type 2; OPN, osteopontin; PD-1, programmed death; pDC, plasmacytoid dendritic cells; PD-L1, programmed death-ligand 1; PIs, proteasome inhibitors; RANK, receptor activator of NF-κB; Treg, regulatory T cells; VEGF, vascular endothelial growth factor.

MM development is conditioned and supported by a wide range of cell types, namely BM stromal cells (BMSC), adipocytes, endothelial cells, osteoclasts, neurons and immune cells. Through soluble factors and/or direct contact, myeloma cells can modulate the phenotype of surrounding BM cells to evade immune surveillance, building a protective environment that confers growth advantages and resistance to therapeutic agents [5,19,20]. Hence, many approved therapeutic strategies can act directly on myeloma cells, or indirectly, by targeting other cell types of the microenvironment, including immune cells, osteoclasts or endothelial cells (Figure 1).

Immune evasion is a hallmark of MM [21,22]. This includes a decrease in B cells with altered differentiation and antibody response [23], and an upregulation of inhibitory molecules either by myeloma cells, antigen-presenting cells (e.g., programmed death-ligand 1 (PD-L1)), or lymphocytes (e.g., PD-1) [24,25]. Furthermore, the presence of tumor-associated macrophages with higher levels of CD163 or CC motif chemokine receptor 2 (CCR2) has also been associated with worse prognosis and drug resistance [26,27]. The impairment in immune homeostasis led to the development of novel therapies that significantly improved MM patients’ survival in the last decade by inducing both tumoricidal and immune-boosting effects [28]. These agents include immunomodulators (IMiD) and, more recently, cereblon E3 ligase modulators (CelMoDs), proteasome inhibitors (PI), monoclonal antibodies (mAb), antibody drug conjugates and chimeric antigen receptor (CAR) T cells. Although not yet approved in MM, other agents disrupting the crosstalk between myeloma cells and the BM microenvironment are also being tested, such as immune checkpoint inhibitors and CAR-NK cells [29,30,31].

The inhibition of osteoblast function and the activation of bone-resorbing osteoclasts are also found in the MM BM milieu [32,33]. This imbalance can be mediated directly by myeloma cells via the receptor activator of NF-B ligand (RANKL), hepatocyte growth factor (HGF) or interleukin (IL)-3, or indirectly via BMSC through the upregulation of RANKL and secretion of IL-6, IL-1β or tumor necrosis factor (TNF)-α [34]. While PIs such as bortezomib have shown to both inhibit osteoclast differentiation and stimulate osteoblast formation [35,36,37], the effect of IMiD is mostly known to affect osteoclastogenesis [38,39,40,41]. Nonetheless, existing reports suggest that combined therapies (e.g., lenalidomide, bortezomib and dexamethasone) may help to induce bone formation [42,43,44]. Moreover, both zoledronic acid, which is a bisphosphonate, and the mAb denosumab are approved for the prevention of osteolytic lesions [45].

The secretion of pro-angiogenic factors has also been associated with MM progression and poor prognosis. Indeed, several research groups have reported an increase in the secretion of the vascular endothelial growth factor (VEGF), hypoxia-inducible transcription factor (HIF)-1α, fibroblast growth factor (FGF), osteopontin (OPN) or angiopoietin (Ang)-1 by myeloma cells. These signals can happen between myeloma cells, BMSC and endothelial cells. Although pre-clinical tests using anti-angiogenic agents against these specific factors have shown promising results, their translation is still not a reality due to relapse and a limited increase in progression-free survival [46,47,48]. Nonetheless, other already approved MM therapies are also reported to target angiogenesis, such as IMiD, PIs or bisphosphonates [49,50,51,52,53].

New axes of development involve the CXC chemokine receptor (CXCR) 4 [54]. For instance, the therapeutic inhibition of CXCR4 (AMD3100) disrupts the interaction between myeloma cells and the BMSC, re-sensitizing myeloma cells to the PI bortezomib, leading to tumor reduction in vivo [55].

Adipocytes may represent another critical regulator of myelomagenesis by protecting myeloma cells from chemotherapy-induced apoptosis [56,57]. The production of fatty acid-binding protein (FABP) 4 by adipocytes upregulates the energy and metabolism of myeloma cells, making the BM microenvironment more pro-tumoral [58]. Preclinical studies strongly suggest that targeting adipocytes or adipose-derived soluble molecules is a worthwhile strategy to tackle MM and/or overcome chemotherapy resistance.

The development of new therapeutic strategies is challenged by serious side effects and/or unreproducible responses due to immune signature, microbiota, specific mutations and patient inter-variability [59,60,61,62]. The discrepancy between the results obtained in pre-clinical trials and clinical outcomes can be attributed to the limitations of the classic tissue culture models, which fail to successfully recreate the whole MM BM niche [8,63,64]. Indeed, MM should be considered a complex ensemble of cells, and over-simplified models are unlikely to recapitulate the complexity of the disease. In the next section, we will address the main limitations associated with current 2D cellular and animal models.

## 3. Historical Evolution, Limitations of 2D Cell Culture and Animal Models

Cell culture represents an indispensable tool for studying cancer cell biology and drug response and has come a long way since the first HeLa cell line was established in 1951 [65]. Scientists can now culture many cell types, including immortalized cancer cell lines, immune cells and even primary human cells [66,67].

Conventional 2D cell culture offers many benefits, including low cost, high-throughput screening capability, standardization and reproducibility [68,69]. However, this technique grows cells on plastic surfaces, resulting in remodeling of the cytoskeleton, altered gene expression and protein synthesis [70]. Studies in planar cell culture have shown how cells become progressively flatter, divide abnormally and lose their differentiated phenotype [71,72]. Two-dimensional MM models mostly use cell line monocultures, which have lost their BM dependence. These do not accurately represent tissue architecture or the interplay between cells, and lack the personal heterogeneity aspects of the disease [4,13]. Transcriptomic studies demonstrated that myeloma cell lines only reflect a limited segment of the whole in vivo profile of the tumor, making them unreliable preclinical models [73]. These also fail to reproduce nutrients and oxygen gradients, limiting the ability of precisely predicting drug sensitivity [74]. The aberrant metabolic profile of MM cells is also a key feature that should be considered. Metabolic pathways provide the energy required for cell proliferation and tumor growth, and are involved in drug resistance events [75,76]. Moreover, the extracellular environment has a major impact on cell structure, mechanic transduction and signaling [77]. In 2D culture, there is a lack of natural extracellular matrix (ECM) proteins, chemokines, growth factors and sites for cellular adhesion, which are crucial for cells to interact with adjacent cells and surroundings, preserving the specificity and homeostasis of the original tissue and its regular functioning [78,79].

Animal models, mostly murine models, provide advantages over 2D models, as the tumor is surrounded by a microenvironment, reproducing tumoral complexity. MM mouse models have shown to be relevant for the recreation of several 3D features of the BM. The 5TMM mice model has been a milestone for the comprehension of MM pathogenesis [80], and the implantation of human fetal bone chips in SCID mice has allowed an even better resemblance of BM physiology and the microenvironment importance in MM [81]. However, mouse models fail to accurately recreate human disease conditions. For example, immunodeficient mice impair the study of immune and cancer cells’ interactions in the microenvironment. Moreover, murine and human tumor microenvironment and immune systems vary significantly. Discrepancies in both innate and adaptive immunity include the balance of leukocyte and immunoglobulin subsets, B-cell and T-cell signaling pathway components, cytokines and cytokine receptors, Th1/Th2 differentiation, the antigen-presenting function of endothelial cells, and chemokine and chemokine receptor expression [82]. Humanized mice could solve this problem, allowing reconstitution of the human immune landscape and cancer–stroma interactions [12]. Nonetheless, studies have highlighted troubles with graft-versus-host disease [83]. Interestingly, Meyer et al. developed patient-derived tumor xenograft models of human acute lymphoblastic leukemia and acute myeloid leukemia, and reported that the murine environment selected specific subclones, resulting in a number of different sub-models [84]. Moreover, this is an expensive time- and resource-consuming approach. Taking into account the timeframe for drug testing, results may be obtained after the patient has suffered mutations and/or developed metastasis, compromising the effectiveness of the selected treatment regimen [85]. The use of poorly validated animal models and the non-reflection of the genetic heterogeneity of human cancers may also constitute some of the reasons that lead to reduced scientific validity and reproducibility of the in vivo studies in biomedical research [69,86]. Finally, with the increasing emphasis on animal welfare, murine models are also known to involve ethical issues. Accordingly, 3D cell culture models may help to decrease the use of laboratory animals in drug testing, going in line with the 3R principles: Replacing animals with alternative methods, Reducing the use of animals in research, and Refining discomfort, whenever possible [87].

All these aspects reinforce the need for a personalized, biologically relevant, complete and accurate preclinical MM model. The idea of 3D culture was first conceived in 1912 by Alexis Carrel, when this scientist cultured an explant from a chick embryo and maintained it over a period of 3 months [88]. Later, aiming to create a cell culture environment that resembled the human body more closely, Hamburger and Salmon developed a model using a soft agar solution [89]. Over the last two decades, 3D culture methods have been widely used to study biology in multiple cancer types (e.g., lung, liver, brain), giving light to new discoveries in the areas of metastasis, hypoxia, angiogenesis and drug screening [90,91,92,93]. Three-dimensional cell culture models enable cells to maintain their natural morphology and tumor architecture, with a proliferating zone and a quiescent region, with limited oxygen, nutrient and growth factor distribution, which might influence drug response [69,70]. With respect to MM, it has been proven that, for example, cytokine production (e.g., IL-6, IL-11 or HGF) is higher in 3D versus 2D cultures [94]. De la Puente et al. showed that their 3D model promoted myeloma cell proliferation better than 2D systems [95]. Spelat and colleagues’ 3D gel was able to maintain pluripotent stem cells in a G0 quiescent state without inducing proliferation or differentiation commitment, but maintaining the potential to do it [96].

Thus, 3D models are proposed as viable and biomimetic alternatives. They might allow for a deeper understanding of molecular disease events and of multidrug resistance mechanisms, improving the predictive value of drug efficacy and safety. The following section depicts 3D MM BM models that are currently available or under development.

## 4. Three-Dimensional MM Models

### 4.1. The Reconstructed Endosteum–Bone Marrow Model

In 2008, a key progress in 3D MM modelling was achieved by Kirshner et al. with the reconstruction of the human MM BM microenvironment in the “rEnd-rBM” model (Table 1) [97]. Twenty-four-well plates were pre-treated with fibronectin and collagen type I, creating the reconstructed endosteum–marrow junction (rEnd) compartment, and this layer was covered with patient BM mononuclear cells (BMMC) suspended in a gel mixture of Matrigel and fibronectin, creating the recombinant BM (rBM) compartment [97]. Cells were cultured in growth medium supplemented with the patient’s own plasma and kept viable for up to 21 days. Curiously, plasma from healthy donors did not support myeloma cell growth, which reinforces the importance of patient specificity. When compared with the patient BM, the rBM environment showed similarities with natural BM niche architecture. Moreover, this breakthrough enabled the proliferation of myeloma cells, including putative stem cells. This is a very relevant feature, as this cell subset seems to be one of the main subsets responsible for drug resistance and relapse [98,99]. Interestingly, an increase in the total number of clonotypic cells was observed, and cells retained the same chromosomal abnormalities present in vivo, as observed after 15 days of culture. Obtaining the entire stromal compartment is a marked improvement of this model over standard culture methods. It allows the testing of several drugs with different targets and the understanding of how these drugs act, not only on myeloma cells, but also on non-tumoral cells. Furthermore, the interaction with stroma may be enough by itself to influence molecular mechanisms related to drug sensitivity and resistance. Results showed the inability of the system to support populations of CD34+ hematopoietic stem cells, CD20+ B cells, and CD138+ plasma cells without the stromal compartment of the BM, highlighting its importance [100].

More recently, based on the “rEnd-rBM” model, Kirshner established zPredicta Inc., commercializing Reconstructed Bone (r-Bone™) technology. This customizable organ-specific 3D platform includes both hematopoietic and stromal cells, as well as extracellular components, to sustain primary human BM cell survival, proliferation and variety [101]. BMMC from 21 newly diagnosed or relapsed patients with MM were set up in r-Bone and cultures were treated according to the clinical single-agent or combination regimen received by the patients. Subsequently, myeloma cell death was evaluated by flow cytometry. The system successfully predicted the patient’s clinical outcome in 90% of the cases [101]. This technology has already been applied in various medical contexts, growing primary BM cells from healthy donors and from patients with MM and amyloidosis.

Huang et al. adapted the rBM concept with the goal of studying the activation of the signal transducer and activator of the transcription (STAT) 3 pathway in MM in 3D versus conventional 2D cultures (Table 1) [102]. The STAT3 pathway was activated when cells were cultured in 3D but remained inactive in conventional 2D cultures, showing how some MM mechanisms are dependent on the 3D structure. Moreover, inhibition of the STAT3 pathway, using the pharmacological selective inhibitor Stattic, significantly decreased the viability of myeloma cells [103]. Furthermore, susceptibility to the PI bortezomib was increased [102], providing insight on targeting STAT3 in MM treatment. Overall, the obtained results suggest that the 3D environment is crucial for recapitulation of ECM proteins and cytokines’ interactions with myeloma cells. This is valuable because MM is usually characterized by deregulated levels of both pro- and anti-inflammatory cytokines [104].

Adapting Huang’s work, Caillot et al. tested whether the inhibition or overproduction of reactive oxygen species (ROS) could sensitize myeloma cells to bortezomib [105], as these cells are known to produce high levels of ROS [106]. Upon modulation of the redox balance, apoptosis and autophagy was increased in myeloma cells [105], suggesting that this may be useful in relapsed/refractory patients, partially reversing tumor microenvironment-mediated drug resistance. Nonetheless, the inclusion of other components of the BM microenvironment would be of interest. Moreover, further characterization of the primary myeloma cells embedded is needed, as most assays were conducted using MM cell lines.

### 4.2. 3D Matrigel Matrix-Embedded Models

Using Matrigel and fibronectin to form a 3D matrix, Perez et al. studied myeloid-derived suppressor cells [107], whose frequency is commonly increased in myeloma niches [108]. BM samples from MM patients were cultured, and granulocytic cell subsets were identified by flow cytometry before and after exposure to the mAb daratumumab [107]. Cultures were maintained for 10 days, and no differences were seen in vitro in the percentage of granulocytic subsets between timepoints of drug exposure.

Cucè and colleagues used a Matrigel–spheroid model to evaluate apoptosis, cell cycle, and changes in cytokine production and release, both in MM cell lines and patient-derived primary myeloma cells (Table 1) [109]. Cultures were exposed to increasing concentrations of trabectedin both in 2D and 3D monoculture or co-culture with monocytes from healthy donors to understand the impact on the nucleotide excision repair pathway [109], previously described to be dysregulated in MM [110]. Trabectedin was able to alter the pro-inflammatory cytokine network, reducing monocyte chemoattractant protein (MCP)-1, VEGF and IL-10 [109]. The authors verified that the presence of monocytes was of utmost importance to recreate the protective effect of the microenvironment on myeloma cells, since 3D Matrigel–spheroid co-culture promoted myeloma cell viability and proliferation in the presence of trabectedin. Spheroids are of particular interest to cancer researchers because they can include heterogeneous cell populations with both proliferating and quiescent cells, owing to limited oxygen and nutrient transport [9,70]. Being scaffold-free, these models take advantage of the natural ability of the cell types to self-aggregate and secrete their own ECM over time [69]. Nevertheless, spheroids have important limitations because they grow as independent cellular aggregates and have reduced interactions with the extracellular setting [111]. Still, if combined with more recent techniques, such as microfluidics or 3D bioprinting, scientists could be able to develop more physiologically relevant 3D models of the disease. These approaches are further described in Section 4.3 and Section 4.7

Very recently, Khan and others put their efforts into developing a 3D platform with the best homology possible to the native hematopoietic tissues, thus allowing the engraftment and growth of primary cells from patients with blood cancers, including MM (Table 1) [112]. For that, differentiated human induced pluripotent stem cells (iPSC) were embedded in a hydrogel with an optimized composition—Matrigel and type I and type IV collagen—in order to support differentiation of the 3D BM perivascular niche [112]. Organoids were obtained and contained hematopoietic stem and progenitor cells, as well as stromal and myeloid cellular subtypes, with homology to human BM cellular subtypes, as confirmed by comparison of the molecular profiles identified by single-cell RNA sequencing. Moreover, cell–cell communication and a vascular network were observed. Interestingly, myeloma cells isolated from BM aspirates of MM patients engrafted into organoids presented a viability of more than 90% at day 10 of culture, whereas in 2D culture, they started to die after only 2 days. These results confirm that patient-derived MM cells require a supportive niche to proliferate and stay viable in ex vivo conditions. This system addresses a major problem of most current models: the vascularization. However, it does not include key populations, such as lymphoid or osteoid cells. Mesodermal, endothelial and hematopoietic lineages were obtained by differentiation of a human episomal cell line and not primary differentiated cells, which can lead to a suboptimal mimicking of the BM physiology.

Using Matrigel as the scaffold also, Braham et al. cultured primary myeloma cells with mesenchymal stem cells (MSC) and endothelial progenitor cells, to facilitate their survival and proliferation, over a 28-day period (Table 1) [113]. Their aim was to investigate the use of a novel class of αβ-engineered T cells to express a defined γδ T-cell receptor (TCR), called TEG, in targeting and eliminating primary myeloma cells. These TEGs migrated inside the Matrigel matrix, finding and killing their targets after 48 h [113]. The 3D model was far more effective when compared to a similar 2D approach. The same 3D model was later employed to test liposomal delivery of drugs (e.g., doxorubicin, bortezomib) [114]. Additionally, it was adapted by other groups to test the efficacy of γδ TCR anti-CD3 bispecific molecules in redirecting T lymphocytes against myeloma cells, while leaving healthy tissues intact [115]. The authors exploited this system to provide pre-clinical in vitro testing of different therapies on primary BM samples from seven relapsed/refractory MM patients [116]. Interestingly, the responses of each donor cell to the given therapies were analyzed according to two different readout parameters (percentage of dead myeloma cells and number of live myeloma cells), resulting in different outcomes. This reflects the importance of selecting the best predictor of clinical response. Nevertheless, the model showed a very poor agreement between in vitro and clinical treatment responses to IMiD. This is most likely due to the lack of events’ recapitulation within the BM microenvironment, such as angiogenic and inflammatory processes, possibly because of incomplete representation of BM cellular subpopulations. Braham et al. also used a bioprinting method with bioactive and biocompatible calcium phosphate cement disks to create separate but interacting endosteal and perivascular subniches of the MM BM, representing an interesting option for studying myeloma–bone interactions. The 3D BM model with combined subniches significantly increased the proliferation of CD138+ myeloma cells [117]. However, even though this is a more complete model, it still does not fully represent the whole BM microenvironment as it lacks, for instance, immune system interactions.

### 4.3. Bioprinted BM MM Models

Cell bioprinting is an emerging approach for 3D cancer cell patterning that simplifies the control of spatial and temporal distribution of cells in a biocompatible material [118]. In 2019, Rodriguez et al. developed a bioprinted MM organoid model using a hydrogel scaffold that combined fibronectin, denatured collagen (Gelin-S), a photo-initiator and a crosslinker, with primary BM aspirates from MM patients (Table 1) [119]. Three-dimensional bioprinters were programmed to extrude 200,000 cells per well in a 96-well plate. Organoids were maintained with a viability above 70% long enough to assess chemosensitivity after one week [119]. This was the first MM organoid model produced using high-throughput bioprinting.

More recently, Wu et al. co-cultured myeloma cells with HS-5 stromal cells in a coaxial extrusion bioprinted construct that consisted of a stiff mineral outer alginate layer and a soft hydrogel core (Table 1) [120]. The authors showed that patient-derived myeloma cells were maintained with good cell viability for up to 7 days, compared to 5 days in the 2D environment [120]. Interestingly, 2D-cultured cells showed higher toxicity to bortezomib than 3D cultures, pointing to the potential improvement in physiological relevance of 3D platforms. Although promising, this model encompasses a low cellular diversity, hindering real mimicking of BM cellular interactions. Common limitations of this method include slow printing speed and the challenge of developing nontoxic bioinks. Although this is still an underexplored approach in the field of MM, its microscale resolution, high precision in forming 3D constructs, and ability to use multiple materials point to the potential of the technique, especially if using the right biomimetic compatible matrix and/or combined with techniques such as microfluidics, addressed further ahead [121,122].

### 4.4. The 3D Tissue-Engineered BM Model

Contributing to another key advancement, de la Puente et al. developed a 3D tissue-engineered BM (3DTEBM^®^) model that consisted of a mixture of myeloma, endothelial and stromal cells from the BM of MM patients (Table 1) [95]. Fibrinogen naturally found in the human plasma and calcium chloride were added for promoting clotting and the crosslinking reaction. Tranexamic acid was added to provide stability to the structure for up to 7 days [95]. Proliferation rates of myeloma cells increased 250% when co-cultured with stromal and endothelial cells, compared to monoculture. Proliferation rates of myeloma cells in the 3DTEBM^®^ were also compared with conventional 2D multi-culture and commercially available 3D models, but these failed to sustain myeloma cell growth, showing the importance of recreating a 3D biomimetic environment. Notably, 3DTEBM^®^ allowed the recreation of drug and oxygen gradients, as suggested by the upregulation of HIF-1α and proto-oncogene serine/threonine-protein kinase (PIM) hypoxia markers, downregulation of CD138 and overexpression of CXCR4 in deeper areas of the scaffold. In addition, the drug uptake measured in cells demonstrated an inverse correlation with depth. Thus, this model seems to be able to recreate the conditions observed in vivo, with two distinct regions, where cells in proximity to vasculature are more proliferative and sensitive to therapy than those in the endosteal niche. This is an important achievement, as it can influence the prediction of resistance to delivered drugs [123,124,125].

Due to its impressive results in resembling the BM niche, the team further explored this model. For instance, they tested the potential of targeting CD47 for immunotherapy [126]. Results showed that when CD47-expressing MM cells were co-cultured with macrophages and treated with an anti-CD47 mAb, a significant killing of MM cells was seen in the 3DTEBM^®^, but not in classic 2D cultures. Extensive motility of macrophages during the phagocytosis process was observed thanks to the hydrogel-like structure of the 3DTEBM^®^, which faithfully simulates the in vivo MM BM niche, in opposition to the adherent nature of 2D cultures. Most recently, the 3DTEBM^®^ model was used to assess different therapies on 19 primary patient samples and found a 89% predictable rate when comparing in vitro results to clinical outcomes [127]. These remarkable drug efficacy findings obtained using the 3DTEBM^®^ technology suggest that this is a highly promising approach for studying primary BM malignancies and those of hematologic origin, such as MM and acute myeloid leukemia.

### 4.5. Hydrogel-Based MM BM Models

Jakubikova et al. used PuraMatrix™ (Becton Dickinson, Franklin Lakes, NJ, USA), a synthetic self-assembling peptide hydrogel (Table 1) [128]. BMMC were obtained from BM aspirates and the adherent fraction containing MSC was expanded for several days before seeding to promote a higher degree of similarity with the in vivo tissue [128]. Cultures were maintained for up to 21 days. A higher expression of both cytokines and ECM molecules known to promote in vivo stem cell expansion and MM survival was observed in the 3D MSC model. These included IL-6, IL-8, MCP-1, VEGF, and collagen I, collagen IV, fibronectin and laminin, respectively. Protein expression analysis comparing 3D and 2D models revealed activation of osteogenesis and osteoclastogenic differentiation, as shown by the upregulation of matrix metallopeptidase 13, osteopontin, and matrix Gla protein and by the downregulation of calmodulin 1 and CXCR4. They also tested a wide range of novel and conventional anti-MM drugs. Particularly, using BM samples from MM patients, sensitivity results between 3D and 2D cultures showed that the drug response in the 3D co-culture model paralleled clinical resistance. Although promising, a higher number of patients are needed to validate these results. Authors report that prospective trials are being conducted to assess the model value in testing personalized immune therapies in MM [128].

Regarding a different approach, Waldschmidt et al. established a conical agarose 3D platform for the propagation of primary BMMC ex vivo (Table 1) [129]. First, myeloma cells growth in the system were compared to a 2D monolayer culture. In 3D, myeloma cells showed a slower initial proliferation, but maintained a more stable growth after 12 days of culture [129]. When comparing 3D monoculture and co-culture conditions, primary myeloma cells did not expand in 3D monoculture, requiring co-culture support by a human stromal cell line (e.g., HS-5, MSP-1). Similar to other groups, detailed cytokine characterization within the developed 3D model revealed a high expression of pro-angiogenic and pro-inflammatory cytokines, such as IL-6, IL-1β, IL-8 and TNF-α [95,128]. This secretion seemed to be stimulated by the interaction of myeloma cells with a stromal co-culture partner, providing the optimal conditions for tumoral cell proliferation and allowing the maintenance of cells in culture for 21 days. Obtained results are in line with studies where newly diagnosed MM patients’ cytokine profiling revealed high levels of IL-6, IL-8 and TNF-α when compared to healthy controls [130]. Therefore, such cytokines seem to be appealing targets for treating MM, and tests can be conducted using the aforementioned platforms. Drug sensitivity tests with bortezomib and auranofin also showed that less toxicity was induced under 3D versus 2D condition and in co- versus monoculture [129,131]. Together, these results point to the important role of stromal co-culture for the in vitro modeling of drug resistance events and to correctly determine the efficacy of therapeutic compounds. From a technical point of view, this conical agarose 3D model shows some advantages, as it allows for clear-cut observation and monitoring by confocal microscopy, flow cytometry or Western blot [132].

Hyaluronic acid (hyaluronan or HA) hydrogels also represent a reliable alternative. HA is an increasingly popular biologically derived matrix, due to its abundant presence in the natural ECM. HA is easier to chemically manipulate compared to other matrix molecules and it allows for the activation of a variety of pathways, including those affecting cell adhesion and motility, inflammation and drug sensitivity [133]. Although being tested in several types of cancer, namely those of the brain, breast and liver [134,135,136,137], few data are available in MM. In 2014, Narayanan et al. developed a 3D HA-based model and showed how matrix composition and stiffness can impact results obtained (Table 1) [138]. In this study, HA-based hydrogels were developed for encapsulating BMSC and myeloma cells. The percentage of survival of myeloma cells grown on medium-stiffness (≥90%) hydrogels was higher than those grown on low- or high-stiffness hydrogels (70–80%) [138]. Proliferation was also higher in medium-stiffness HA hydrogels when compared to 2D or Matrigel cultures. As is well-accepted, during cancer, cells are subjected to a variety of mechanical changes in their microenvironment, resultant in part from ECM remodeling, with events such as increased crosslinking activity and deficient matrix degradation [139]. These events lead to increased matrix stiffness, which in turn activates transcription factors that promote tumor cell proliferation and growth [140,141]. A study conducted with monoclonal gammopathy of undetermined significance and MM patients showed that altered ECM protein expression (e.g., osteopontin, periostin, entactin and fibulin) is associated with decreased overall survival [142]. Therefore, using 3D models for studying proteins that are involved in matrix crosslinking and stiffness management may help identify new targets, if found to be dysregulated in MM.

### 4.6. Dynamic MM BM Models

Some characteristics cannot be assessed in static culture approaches, such as circulation of nutrients, waste removal and sheer forces. Thus, in 2013, Ferrarini et al. first adapted bioreactor technology (Rotary Cell Culture System (RCCS^TM^), Synthecon Inc., Houston, TX, USA) to grow primary myeloma cells (Table 1) [143]. This rotary cell culture system allowed the creation of a dynamic model in which cells are in a “free fall” form, favoring mass transfer of nutrients and low shear stress conditions [143,144]. On a first approach, this was designed for culture tissue explants from MM patients, with well-preserved tissue architecture and cell viability. Through histology, it was possible to observe that myeloma cells were kept viable within their microenvironment, as well as the vessels that appeared disrupted in static culture. Assessment of β2-microglobulin levels in supernatants from bortezomib-treated samples and in patients’ sera after bortezomib-based therapy showed an overall agreement in the drug response ex vivo and in vivo. However, there were issues with tissue harvesting and reproducibility: (1) the obtained biological samples are very sensitive to manipulation, requiring careful handling to preserve integrity; (2) the number and size of the tissue fragments need to be thoroughly determined to counterbalance the hydrodynamic forces generated in the culture chamber and maintain the free fall condition; and (3) for different conditions tested in parallel, each culture vessel needs to have the exact amount of samples and comparable weight and volume [144].

In a follow-up study, the same group focused on isolated primary myeloma cells (Table 1) [145]. These were co-cultured with allogeneic BMSC and human umbilical endothelial vein cells (HUVEC), supporting the survival of myeloma cells for up to 7 days, in the bioreactor. Of note, genomic analysis was performed in a MM patient and compared to the bioreactor culture, showing that it paralleled the expansion of the clone that dominated in vivo [145]. Interestingly, signaling pathways associated with tumor survival, proliferation and drug resistance were upregulated in the 3D bioreactor model when compared with conventional 2D models. This indicates that this 3D model can be useful for studying drug resistance and its association with specific genomic alterations. Overall, this construct allowed for the reproduction of several MM characteristics and tumor–stroma interactions. The use of allogeneic BMSC in co-culture with patient-derived myeloma cells can be considered a strength of this study when compared to the models developed using stromal cell lines (e.g., HS-5 [105,120,131]). However, one could wonder if using both autologous BMSC and HUVEC would allow for a better recapitulation of the patient’s BM niche specificity.

### 4.7. 3D Models of Myeloma–Bone Interactions

Despite carrying advantages, the rotary system model requires sophisticated devices [146]. A more affordable approach includes microfluidic devices. Microfluidics seems to be a promising technology for 3D cell culture as it brings the possibility of creating biomimetic structures in which biological processes can be recapitulated [147], including circulation and cellular compartmentalization, in a controlled physiological environment [148,149]. In the last number of years, this micromanipulation technique has been employed in efforts to develop a system that allows for the easy placing of cells and matrices into the culture chambers of the device and supports replication and characterization of tissues and tumor microenvironments at a low cost [150].

Zhang et al. focused on the continuous nourishment of the cells, attempting to replicate the body’s circulation in an 8-chamber microfluidic device (Table 1) [151]. Primary BMMC were introduced in each chamber after generation of an ossified tissue scaffold from a human osteoblastic cell line (line hFOB 1.19), resembling the endosteal surface, to mimic interactions between the two cellular populations [151]. Real-time monitoring confirmed that myeloma cells expanded after 21 days in culture and were drawn towards the osteoblast layer. The group also compared cell viability and proliferation of cells in their model to a 3D static culture, showing that perfusion in the endosteal niche is a key factor of the microenvironment and improves maintenance of long-term culture of myeloma cells [152,153]. However, the authors showed concerns regarding the microfluidic device material polydimethylsiloxane, as this has been reported to leach hydrophobic components from cell culture media, including drugs, antibodies and growth factors, potentially affecting the system’s reproducibility, which is essential for validation steps and clinical testing [153]. Moreover, this model is based on a simple co-culture of myeloma and bone-derived cells. Because a monolayer of osteoblasts is cultured on the surface of the microfluidic device, and then myeloma cells are pumped into the chambers, this methodology raises the question of whether a 3D scaffold should be considered here. The addition of multiple patient-derived BM components would be of interest to increase the quality and complexity of the model, paving the way for relevant studies of cell adhesion-mediated drug resistance, for example.

In this context, Reagan et al. attempted to recreate the BM microenvironment by stimulating MSC to undergo osteogenic differentiation inside porous silk scaffolds prior to the inclusion of myeloma cells (Table 1) [154]. Cell viability after bortezomib treatment was determined, showing drug resistance in their 3D culture, when compared to hydrogel and 2D cultures [154]. These findings suggest that the inclusion of bone cells and recreation of a bone-like component are important factors for cell adhesion-mediated drug resistance. Additionally, the authors were able to use this model to identify a new microRNA signature associated with dysfunctional osteogenesis of MSC in contact with MM cells [154], showing that 3D models can be used to identify novel biomarkers and potential new therapeutic targets [155]. These 3D silk scaffolds have also been employed to develop the first 3D tissue-engineered BM adipose tissue (BMAT) model. Briefly, MSC were seeded in silk scaffolds and cultured with adipogenic media, driving differentiation of adipocytes. With this approach, Fairfield et al. were able to shed light on complex interactions between BMAT and tumor cells, even if no patient-derived cells were used [156]. Compared with 2D cultures, pathways of DNA replication, metabolic and proliferation events were upregulated in BM adipocytes in this model. Of note, a study combining in vitro and in vivo methods showed that BM of MM patients contained, in fact, an increased number of both preadipocytes and mature adipocytes, pointing to its potentiality as a target and to the value of using a 3D model that includes cells of the adipose tissue in culture [157].

### 4.8. Computational and Mathematical MM BM Models

Over the years, mathematical models have been developed to simulate tumor growth and to study tumor dynamics in response to therapy and drug resistance [158,159]. Although these models are very powerful tools for the analysis of complex interactions within the tumor microenvironment, they are often derived from experimental conditions that are very controlled, hindering their translation to the clinical practice.

In 2014, Khin et al. combined in vitro microfluidics and in silico approaches to assess drug response in MM. Myeloma and stromal cells from 10 MM patients were cultured in 3D culture slides with collagen type I, and the effect of bortezomib and melphalan was tested [160]. An algorithm of digital image analysis detected the number of live myeloma cells by the motion of the cell membrane for different drug concentrations at different timepoints. The viability measurements obtained in vitro were fitted to the mathematical model of chemosensitivity, representing one or two subpopulations, with specific size, doubling time and level of sensitivity to the drug. Data derived from these experiments were then used to parameterize mathematical models for clinical outcome simulation [160].

This model was improved through the development of an ex vivo platform capable of making a three-month prediction regarding clinical response [63]. The Ex vivo Mathematical Myeloma Advisor (EMMA) combined a digital image analysis algorithm, mathematical models and pharmacokinetic data to predict the effect of 31 chemotherapeutic agents and IMiD. Primary myeloma cells were seeded with human BMSC in medium supplemented with patient-derived plasma. A digital microscope snapped live images, which were gathered every 30 min over 4 days. Nonetheless, the described system was unable to quantify cellularity of adherent cancer cells [161]. For the characterization of tumor heterogeneity with different degrees of chemosensitivity, the group considered and tested four hypotheses: (1) the existence of only one clonal population in which all tumoral cells presented the same degree of sensitivity to a particular drug; (2) the existence of only one population, but with a chemosensitivity following a normal distribution; (3) the existence of two clonal subpopulations; or (4) the existence of two normal distributions [63]. This may be advantageous because patients presenting heterogeneous tumors can exhibit clones that are more resistant to therapy. Even though more cells of the BM microenvironment are not present in the model (e.g., immune cells), the results obtained with this approach were very promising.

Nonetheless, the use of EMMA for drug combinations revealed some flaws: predictions of response to therapy were generated by simulating each of the drugs independently and combining all responses, assuming additivity [63]. In 2020, the same group extended EMMA into a synergy-augmented model (SAM), which captured interactions between drugs, transforming the fixed combined effects of the in vitro drug concentrations into clinically relevant, time-varying ones [64]. The modified damages caused by a drug when in the presence of another drug was considered and deemed to accumulate over time. Cell death happened when either drug exceeded the corresponding tumor-specific threshold. To choose the model that best represents data of tumor heterogeneity, the Akaike information criterion is applied, balancing the goodness of fit with the complexity of the model [162]. Then, tumor drug-specific parameters are combined with pharmacokinetic data, simulating patient-specific clinical responses to each drug. To do so, tumor growth must be estimated. “Median lethal dose” (LD50) and “area under the curve” are metrics that cannot handle pharmacokinetic/pharmacodynamic complex relations [63,64]. For that reason, in this context, the best response is predicted based on clinical response parameters (from EMMA and SAM models), combined with pharmacokinetic data. Still, we cannot fail to note that this model was developed without the use of a validation cohort [63]. Although the methodology used does not require a different group, it would be a great asset to validate the results in a completely independent cohort.

EMMA was built considering a cohort of 52 MM patients. This model correctly classified 50 out of 52 patients (96%) according to response/no response to treatment, and demonstrated that 60% of the patients received at least one drug with no clinical efficacy [63]. For the improved SAM model, patient-specific parameters were estimated by fitting ex vivo drug sensitivity data of 203 patients with pharmacokinetic data [64]. The two-drug combination effect was evaluated for 46 different combinations and their synergistic or antagonistic relation was assessed, and only 4 combinations were considered to be clinically synergistic [64]. Remarkably, this result shows that the synergism observed in the bench does not always translate into the bedside, referring to the benefit of combinations over single-agent regimens mainly due to independent drug action.

Although there is no simple formula for building the perfect model, there are some characteristics that we consider essential in obtaining a system that is both representative and easily applied in clinical practice. In general terms, the mathematical/computational approach needs to extrapolate in vitro predictions into a clinical outcome. To this end, the effect that different drugs have at the cellular level, not only singularly but also when combined, needs to be accounted for. Drug concentration, drug-induced damage and tumor growth equations are required. When in the presence of a combined therapy regimen, defining a dose–response function becomes more complex, since the synergetic effect must be considered, requiring, in addition to the effects of single agents, the inclusion of functions that define the effect due to the interaction of the drugs. Importantly, most patients are treated with triple therapies [163]. Therefore, testing the combined effect of more than two agents should be the next step to obtain a more accurate model. Nonetheless, this will significantly increase the complexity of the mathematical model. Additionally, parameters such as drug-induced repair rate and the drug-induced cell damage effect must also be included, as well as microenvironment-specific parameters regarding the immune compartment, matrix stiffness, and assessment of changes/effects in cells other than myeloma cells. Finally, the inclusion of -omics parameters (e.g., proteomics, genomics, transcriptomics), also possible to assess in vitro [164], must be evaluated, as they allow for even greater customization of the model to each patient’s characteristics.

## 5. An Eye into the Future—Challenges and Solutions

While 3D cell culture allows a more accurate representation of the natural environment of cells, these models also present new difficulties. One of the most important challenges to overcome concerns the ability to obtain and grow primary cells, maintaining their viability, integrity and phenotype. Primary cells usually take time to adapt and easily become senescent [165]. Therefore, time is a key factor, as tests must be conducted before the growth potential of primary cells is exhausted. Preferably, the culture should be carried out using freshly harvested biopsy samples. However, they are not always available, even for research groups that have a close link to medical institutes, which makes it difficult to develop, optimize and validate new assay platforms.

The obtained sample usually needs processing, either for isolation of specific types of cells or for removal of others (e.g., erythrocytes), introducing additional variations in tissue architecture, microenvironment and sample composition. Hence, we believe that it is of the utmost importance to take benefit of the whole sample, or as much as possible, to avoid intense manipulation and ensure the presence of all the different cell types, including stem, immune and bone cells. This will enable the study of their molecular mechanisms and drug susceptibility.

Co-cultures increase the complexity of the model and, therefore, require optimization of both cell ratios and cell media composition (including medium supplements specific to the needs of the cells in culture, such as growth factors or patient-derived plasma) to allow the proper growth of all cell types in terms of biological and disease relevance. Renewal of the medium is also an important aspect to consider: On one hand, the procedure must be done carefully to avoid disruption of the scaffold. On the other hand, the amount and frequency of medium renewal need to be carefully considered and adjusted according to each case, since the process might remove factors secreted by the cells that are most likely essential for their maintenance and can interfere with physiological behavior. Interestingly, Kirshner’s model can maintain cell viability for 30 days without changing the medium [100]. The authors advise to only change half of the total medium volume at a time if medium renewal is required [100]. Due to the complexity of the immune system, which includes several subpopulations and activation states, co-culturing immune cells in a 3D MM model is a major challenge. Given the increasing interest in cancer immunotherapy, efforts are being made to create models that allow tumor–immune cell interaction [166,167]. It is of utmost importance to make sure that immune cell effector responses are not affected in 3D culture. Immune cell infiltration (e.g., lymphocytes, macrophages and myeloid cells), activation and cytokine secretion must be evaluated, making sure that cells are able to display their antitumor potential, which is mediated by immunotherapeutic agents [168,169,170]. Interestingly, the work of van Diest et al., using γδ TCR anti-CD3 bispecific molecules in a 3D BM niche model with stromal, endothelial and myeloma cells in Matrigel, revealed the successful infiltration of T cells and selective elimination of myeloma cells in culture [115]. The presence of functional tumor–stroma interactions in a 3D MM model may provide valuable information on therapeutic efficacy and adverse effects of immunotherapies [126,145].

The ideal model should provide the ability to identify key factors regulating tumor development, such as cell–cell interactions, cell–matrix interactions and cell receptors [9]. For that, it must comprise the following aspects: good recapitulation of biochemical cues, adequate tissue stiffness, permeability for penetration of drugs, nutrients and growth factors, recreation of oxygen gradients, sites for cell adhesion, compartmentalization and vascularization, and must allow proliferation, differentiation and migration. Previous experiments have shown how culturing the same cells in different biomaterials leads to changes in the obtained results [138,154]. Hence, selecting the scaffold to be used is of key importance in the experimental process, and should not be disregarded. Although several new options have emerged in the past 30 years, scaffolds of natural origin, namely Matrigel [171], collagen [172] and HA [173], are still the gold standard in cancer research. These are naturally recognized and remodeled by the cells, and possess cytokines and growth factors, which allow for the improvement of viability, while cells carry out their functions [174,175]. However, most of the ECM gels are obtained from animals or cultured cells, leading to batch-to-batch variation that reduces reliability and reproducibility [176]. To avoid such inconsistencies, some alternatives are recommended, such as growth-factor-reduced Matrigel instead of the standard Matrigel, for instance. In addition, these gels often require fast handling and control of temperature or crosslinking activity in order to avoid premature polymerization of the matrix, a challenge in itself [177,178]. Recently, xeno-free hydrogel options are emerging that do not require crosslinking agents and can be manipulated at room temperature [179,180].

Improvements in imaging techniques are also fundamental in making the most out of the third dimension. Confocal microscopy enables visualization of cells in situ but, for now, it is fairly difficult to visualize 3D samples on microscopes due to their thickness and poor light penetration [181]. More transparent matrices are preferable for more straightforward monitoring using non-destructive methods. For example, Waldschmidt et al. state that their agarose hydrogel allows for easy examinations and does not absorb any of the fluorescent dyes incubated to stain cells for fluorescent microscopy, providing a clear background in imaging [129,132]. On the other hand, most 3D models can be fixed and embedded in paraffin, allowing slicing into several sections to be examined. However, even though immunohistochemistry provides a wide range of markers for visualizing cell behavior, it preserves limited spatial characteristics, reducing the 3D model down to 2D [182]. Fixation of the samples is sometimes demanding because some matrices tend to depolymerize with fixative agents and the addition of reagents that minimize this action can increase the imaging background [183]. The right balance between these agents must be found, to avoid compromising the value of the model. Attention should also be paid to the processing of the sample for histological cuts, as certain scaffolds are too sensitive and may tear apart. Notably, the 3D culture of Huang et al. was formed in a well with HistoGel^TM^ that could be easily fixed, allowing for histologic processing and immunocytochemical studies [102,184].

Analyzing 3D samples by flow cytometry can be challenging, as samples must be in cell suspension, requiring dissociation by single or combined enzymes (e.g., trypsin, hyaluronidase, collagenase, dispase, cell recovery solution), which can affect cell integrity and viability. In addition, spatial distribution is lost. Spelat et al. introduced a nice advantage regarding this matter, as their worm-like micelle gel can be converted into free-flowing spheres simply by incubating the culture in a 4 °C solution, therefore avoiding enzymatic degradation, as required by many commercially available protein-based matrix gels [96]. Thanks to technological advances, new flow cytometers are being developed, aiming to allow the analysis and sorting of intact structures up to 1500 μm, instead of cell suspension [185,186].

As for drug screening, the major challenge regards the high tumor heterogeneity, not only at the inter-patient heterogeneity level, as analyses reveal high genomic heterogeneity across patients [187,188]; but also at the temporal and spatial intra-patient heterogeneity levels [74,189]. Anti-tumoral drugs might work out differently in cells at different stages, impacting the guidelines for treatment, and the whole setting of a patient’s disease may not be represented simply by the portion of biologic material collected in the biopsy [190]. Time is also an obstacle because the model needs to be grown and validated before screening. A defined repertoire of drugs to be screened is also required for setting a realistic and convenient timeframe. Interestingly, Braham et al. showed that drug resistance in their model was only seen when administrating treatments 5 to 7 days after assembling 3D cultures. This time was necessary for the supporting cells in Matrigel to spread and form networks, pointing to the need to allow the establishment of myeloma–BM interactions before testing therapeutics [114]. The lack of drug-metabolizing enzymes in an ex vivo context can also impact the evaluation of anti-tumor drug effects in the 3D model. Considering the importance of metabolizing compounds over different tissues of the human body, including the liver and kidneys [191,192], studies using multi-organ systems have emerged. These allow for the establishment of organoids of different origin, linked and communicating on a chip, for integrated responses and more predictive assessments [193,194].

For now, there is still a lack of standardized procedures for creating reliable platforms to be used in personalized medicine. Most of the developed models are complex and recreating them is a major task for other groups, hampering the transition to the drug discovery industry. The ideal 3D MM model should be easy to perform, reproducible, timesaving, cost-effective, and should allow for high-throughput screening while still mimicking the tumor cells’ behavior and their crosstalk with the BM microenvironment. As a result, a greater knowledge of the mechanisms and pathways involved in MM is expected and, consequently, developing drugs capable of engaging with them should become easier [9]. From our point of view, there are some paramount concerns to take into account when trying to assess cells’ behavior and the therapeutic response: (1) Do we have all the necessary cells in culture, both in type and number? (2) Are all the BM compartments replicated? (3) What are the fundamental tumor niche components affecting disease progression? (4) What are the possible drug targets that must be present in culture? (5) What is the endpoint of interest and how do we consider it?

**Figure 2:**
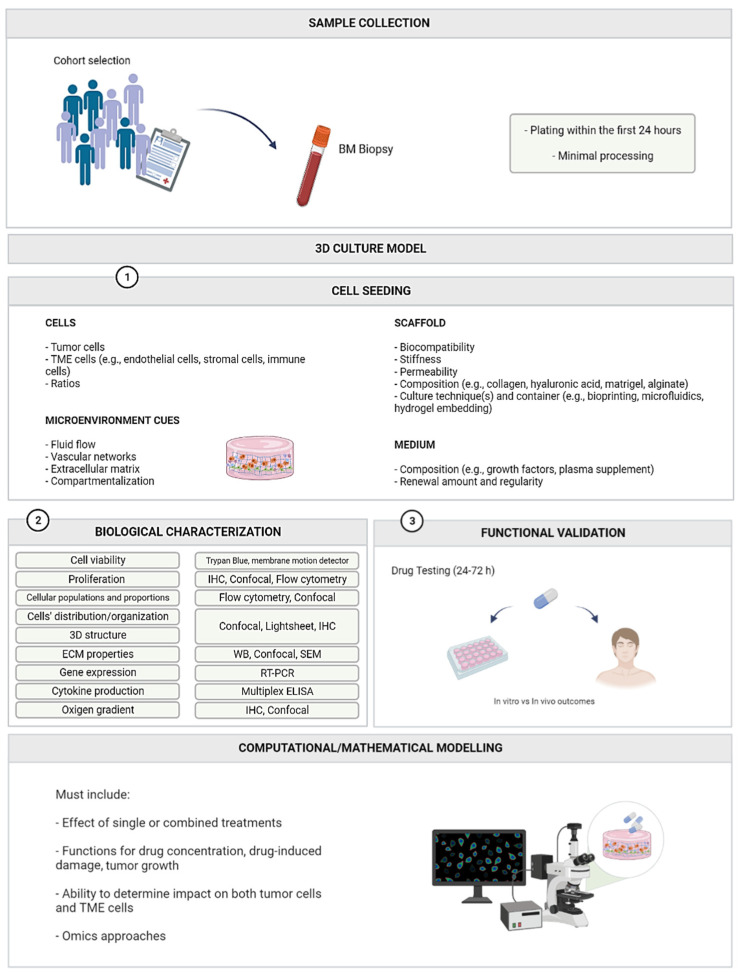
Key points for obtaining the ideal 3D MM model. Even though efforts to create an authentic and reliable MM model are increasing, there are still issues that need to be addressed and improved. Here, we sum up the main factors and variables that are essential and must be considered before, during and after developing a 3D MM model. They include sample processing, culture establishment, biological assays, validation and prediction of clinical outcomes. Created with BioRender.com. Abbreviations: BM, bone marrow; ECM, extracellular matrix; ELISA, enzyme-linked immunosorbent assay; IHC, immunohistochemistry; RT-PCR, reverse transcriptase polymerase chain reaction; SEM, scanning electron microscope; TME, tumor microenvironment; WB, Western blot.

Including emerging technologies in pre-clinical studies such as microfluidics and computational modeling will most likely push 3D models forward in clinical translation (Figure 2). 

## 6. Conclusions

The scientific community is becoming more aware of the importance of having a reliable and representative model of the in vivo scenario of MM. The success of the 3D model’s establishment and therapy response prediction depends heavily on the ability of the model to recapitulate tumors’ biology and physiology as well as their microenvironment. While there are still many challenges to overcome, including finding the right balance between the necessity to achieve the perfect model and the ease of its usage, it seems clear that the ultimate model should be based on patient samples, as every tumor has its own individuality. This would provide a powerful tool for patient-tailored profiling in pathophysiology studies and drug response, potentiating its translation from bench to bedside.

## Figures and Tables

**Table 1:** Available 3D patient-derived MM models. *n* of BM samples, number of samples obtained from patient biopsy and used for cell culture in the 3D model; *n* of patients, number of patients who entered a study of correlation of the obtained laboratory results in 3D culture versus clinical outcome. Abbreviations: BM, bone marrow; BMMC, bone marrow mononuclear cells; BMSC, bone marrow stromal cells; CaCl_2_, calcium chloride; CFSE, carboxy fluorescein succinimidyl ester; CFU, colony-forming unit; ECM, extracellular matrix; FISH, fluorescence in situ hybridization; GelMA, gelatin methacryloyl; HARV, high-aspect-ratio vessel; HUVEC, human umbilical vein endothelial cells; IHC, immunohistochemistry; iPSC, induced pluripotent stem cells; me-HA, methacrylated hyaluronic acid; MM, multiple myeloma; MSC, mesenchymal stem cells; nHA, nanohydroxyapatite; PBMC, peripheral blood mononuclear cells; PDMS, polydimethylsiloxane; PI, proteasome inhibitors; rBM, reconstructed bone marrow; RCCS, rotary cell culture system; rEnd, reconstructed endosteum; scRNA seq, single-cell RNA sequencing; WB, Western blot. * Approved patent.

Model	*n* BM Samples/*n* Patients	Scaffold	Cells	Max. Time in Culture	Cellular Integrity Evaluation	Tested Drugs Ex Vivo Correlation to Clinical Outcomes	References Patent
**The Reconstructed Endosteum–Bone Marrow Model**							
3D Model of Reconstructed Human BM *	*n* = 48 samples *n* = 21 patients	Culture plates coated with fibronectin and collagen I (1:1) (rEnd), overlaid with BMMC in an ECM mixture of Matrigel and fibronectin (2:1) (rBM). 48-well plates	Primary BMMC	>30 days	Cell viability (Flow cytometry), Proliferation (CFU assay), Cell organization in the 3D structure (IHC and confocal microscopy)	Melphalan and bortezomib, 90% predictability (*n* = 21; 6 drugs alone or in combination)	Kirshner et al., 2008 [97]; Parikh et al., 2014 [100]; https://www.zpredicta.com/ (accessed on 20 May 2022) Patent number WO2018023129A1, SG10201805928WA
3D Reconstructed MM Bone Marrow Model	*n* = 15 samples	48-well plates coated with fibrinogen/collagen I. MM cells suspended in Matrigel, fibronectin and collagen IV (4:2.5:1)	Primary BMMC	7 days	Cell viability (Trypan blue exclusion assay), Proliferation (CFSE assay), MM cell percentage (flow cytometry)	IL-6 and Stattic	Huang et al., 2018 [102]; Huang et al., 2021 [103]
**3D Matrigel Matrix-Embedded Models**							
3D MM Organoid Model	*n* = 3 samples *n* = 36 patients	Matrigel and fibronectin (2:1) overlaid with 10% patient-derived plasma medium. 48-well plate	Primary BMMC	10 days	Cell viability and population representativity (flow cytometry)	Daratumumab. Percentage of granulocytic subsets in BM samples similar to corresponding MM patients analyzed before and after treatment	Perez et al., 2020 [107]
MM Matrigel–Spheroids	*n* = 3 samples	Matrigel drop 24-well plate	Primary MM BM cells Monocytes from healthy donors’ PBMC	3 days	Cell viability (flow cytometry and WB of cleaved/total levels of cell death-related proteins)	Trabectedin	Cucè et al., 2019 [109]
Human Bone Marrow Organoid	*n* = 3 samples	Matrigel, type I and IV collagen 96-well ultra-low-attachment plate	Primary MM BM cells iPSC-generated mesenchymal and myeloid cells and vasculature	>10 days	Cell viability (CellVue staining), Cell subpopulation representativity (flow cytometry), Homology of differentiated iPSC to in vivo conditions (scRNA-seq)	No drug tested	Khan et al., 2022 [112]
3D Bone Marrow Niche Model	*n* = 8 samples	Growth factor-reduced Matrigel diluted in MSC-medium (1:1)	Primary BM MM cells BMSC from healthy BM HUVEC	28 days	Cell viability (Live/dead in confocal microscopy)	TEGs and liposomal delivery of doxorubicin and bortezomib. Ex vivo responses to alkylating agents and PI showed good agreement with clinical responses (κ = 0.75). Ex vivo responses to IMiD showed very poor agreement with clinical responses (κ = 0.00 to κ = −0.50).	Braham et al., 2018 [113,114]; Braham et al., 2019 [116]
**Bioprinted BM MM Models**							
High-throughput 3D MM Bioprinted Organoid Model *	*n* = 45 samples	Bioink hydrogel mixture of growth factors, thiol-fibronectin and a crosslinker	Primary BMMC	7 days	Cell viability and population representativity (ATP quantification, IHC and flow cytometry)	Lenalidomide, cyclophosphamide, pomalidomide, bortezomib and dexamethasone.	Rodriguez et al., 2019 [119] Patent number WO2016064648A1
3D Bioprinted MM Model	*n* = 2 samples	Bioink combination of GelMA, alginate, nHA and a crosslinker. Outer mineral-containing sheath, inner soft hydrogel-based core	Primary MM BM cells	7 days	Cell viability (Live/dead staining in confocal microscopy)	Bortezomib and tocilizumab	Wu et al., 2021 [120]
**The 3D Tissue-engineered BM Model**							
3D Tissue-engineered Bone Marrow *	*n* = 19 patients	Crosslinking of patient plasma fibrinogen with CaCl_2_ and tranexamic acid 96-well plate	Primary BMMC	7 days	Cell viability and proliferation (flow cytometry/MMT for impact of tranexamic acid), Oxygen gradient (IHC)	Doxorubicin, bortezomib, carfilzomib 89% predictability (*n* = 19; 10 drugs alone or in combination)	de la Puente et al., 2015 [95]; Alhallak et al., 2021 [127] Patent number US20160136327
**Hydrogel-based MM BM Models**							
Hydrogel-based 3D BM Niche Model	*n* = 52 samples	0.5% PuraMatrix hydrogel, overlaid with medium and MSC	Primary MM BM cells and primary MSC	21 days	Cell viability (flow cytometry), Proliferation (CFSE assay)	Thalidomide, lenalidomide, pomalidomide, bortezomib, carfilzomib, reserpine, doxorubicin, dexamethasone and melphalan. Sensitivity results in the 3D co-culture model paralleled the observed clinical resistance (*n* = 4; 2 drugs)	Jakubikova et al., 2016 [128]
Conical Agarose 3D Co-culture Platform	*n* = 6 samples	Agarose-based microwell disk-shaped device with conical microwells. 6- and 24-well plates	Primary MM BM cells HS-5 stromal cell line	21 days	Multimodal viability assessment (microscopy, panchromatic Pappenheim staining and IHC for CD38), Cell proliferation (ATP quantification and cluster volume), Cytokine expression (multiplex cytokine assay)	Bortezomib and Auranofin	Müller et al., 2017 [132]; Waldschmidt et al., 2022 [129]
Hyaluronic Acid-based 3D Hydrogel	n.d.	Hydrogel discs of photocrosslinkable-meHA and GelMA. 48-well plate	Primary MM BM cells Primary BMSC	21 days	Cell viability (Live/dead or Trypan blue assay), Proliferation (MTT and CFU assay)	No drug tested	Narayanan et al., 2014 [138]
**Dynamic MM BM Models**							
3D Dynamic Culture Model of Tissue Explants	*n* = 5 samples	RCCS-1 bioreactor in 10mL HARV culture vessels	Patient-derived tissue explants (2–3 mm^3^ of maximal volume)	14 days	Soluble factor expression (particle-enhanced immunonephelometry and SDS-PAGE), Microvessel density quantification	Bortezomib. β2-microglobulin levels in culture supernatant similar to levels in patients’ sera before and after bortezomib-based therapies (*n* = 4)	Ferrarini et al., 2013 [143]
3D Dynamic Culture Model for MM Cells	*n* = 7 samples	Gelatin scaffolds with pre-embedded cells included in bioreactor technology	Primary MM BM cells Primary BMSC HUVEC Osteoblasts differentiated from HUVEC	7 days	Cell viability (flow cytometry), Cell proliferation (IHC, genetic profiling (FISH), soluble factor expression (multiplex cytokine assay and zimography)	Bortezomib. Ex vivo anticipation of the in vivo dominant clone expansion in a high-risk MM patient	Belloni et al., 2018 [145]
**3D Models of Myeloma–Bone Interactions**							
Osteoblast -derived 3D Tissue Scaffold *	*n* = 3 samples	PDMS microfluidic chambers coated with human fibronectin. Flow rate of 0.8 mL/min	Primary BMMC hFOB cell line	21 days	Cell proliferation (CFSE), Cell subpopulation representativity (flow cytometry), Interactions of BMMC with the 3D ossified tissue (real-time brightfield and fluorescent imaging)	Carfilzomib	Zhang et al., 2014 [151]; Zhang et al., 2015 [152] Patent number EP2970432A1
3D Silk Scaffold Model of BM Niche	*n* = 4 samples	Porous and aqueous silk scaffold pre-seeded with MSC in osteogenic medium for differentiation into bone	Primary MM BM cells Primary MSC from normal healthy subjects or MM patients	>30 days	Cell viability (Live/dead in confocal microscopy)	No drug tested	Reagan et al., 2014 [154]
**Computational and Mathematical MM BM Models**							
Ex vivo Mathematical Myeloma Advisor (EMMA)	*n* = 52 patients	Collagen type I matrix 384- and 1,536-well plates	Primary MM BM cells Primary BMSC	5 days	Cell viability (presence or absence of membrane motion)	31 novel and chemotherapy agents. 96% predictability (*n* = 52; 31 drugs). Able to predict 3-month clinical response within 5 days	Silva et al., 2015 [161]; Silva et al., 2017 [63]

## Data Availability

Not applicable.

## References

[1] Sung H., Ferlay J., Siegel R.L., Laversanne M., Soerjomataram I., Jemal A., Bray F. (2021). Global Cancer Statistics 2020: GLOBOCAN Estimates of Incidence and Mortality Worldwide for 36 Cancers in 185 Countries. CA. Cancer J. Clin..

[2] Rajkumar S.V., Dimopoulos M.A., Palumbo A., Blade J., Merlini G., Mateos M.V., Kumar S., Hillengass J., Kastritis E., Richardson P. (2014). International Myeloma Working Group Updated Criteria for the Diagnosis of Multiple Myeloma. Lancet. Oncol..

[3] Palumbo A., Anderson K. (2011). Multiple Myeloma. N. Engl. J. Med..

[4] de la Puente P., Azab A.K. (2016). 3D Tissue-Engineered Bone Marrow: What Does This Mean for the Treatment of Multiple Myeloma?. Future Oncol..

[5] Manier S., Sacco A., Leleu X., Ghobrial I.M., Roccaro A.M. (2012). Bone Marrow Microenvironment in Multiple Myeloma Progression. J. Biomed. Biotechnol..

[6] Romano A., Conticello C., Cavalli M., Vetro C., La Fauci A., Parrinello N.L., Di Raimondo F. (2014). Immunological Dysregulation in Multiple Myeloma Microenvironment. Biomed Res. Int..

[7] Hanahan D., Coussens L.M. (2012). Accessories to the Crime: Functions of Cells Recruited to the Tumor Microenvironment. Cancer Cell.

[8] Papadimitriou K., Kostopoulos I.V., Tsopanidou A., Orologas-Stavrou N., Kastritis E., Tsitsilonis O., Dimopoulos M.A., Terpos E. (2020). Ex Vivo Models Simulating the Bone Marrow Environment and Predicting Response to Therapy in Multiple Myeloma. Cancers.

[9] Alemany-Ribes M., Semino C.E. (2014). Bioengineering 3D Environments for Cancer Models. Adv. Drug Deliv. Rev..

[10] Sharma M.R., Stadler W.M., Ratain M.J. (2011). Randomized Phase II Trials: A Long-Term Investment with Promising Returns. J. Natl. Cancer Inst..

[11] Kazandjian D. (2016). Multiple Myeloma Epidemiology and Survival: A Unique Malignancy. Semin. Oncol..

[12] Shultz L.D., Brehm M.A., Victor Garcia-Martinez J., Greiner D.L. (2012). Humanized Mice for Immune System Investigation: Progress, Promise and Challenges. Nat. Rev. Immunol..

[13] Fan H., Demirci U., Chen P. (2019). Emerging Organoid Models: Leaping Forward in Cancer Research. J. Hematol. Oncol..

[14] Vinci M., Gowan S., Boxall F., Patterson L., Zimmermann M., Court W., Lomas C., Mendiola M., Hardisson D., Eccles S.A. (2012). Advances in Establishment and Analysis of Three-Dimensional Tumor Spheroid-Based Functional Assays for Target Validation and Drug Evaluation. BMC Biol..

[15] van de Donk N.W.C.J., Pawlyn C., Yong K.L. (2021). Multiple Myeloma. Lancet.

[16] Palumbo A., Avet-Loiseau H., Oliva S., Lokhorst H.M., Goldschmidt H., Rosinol L., Richardson P., Caltagirone S., Lahuerta J.J., Facon T. (2015). Revised International Staging System for Multiple Myeloma: A Report from International Myeloma Working Group. J. Clin. Oncol..

[17] Dagogo-Jack I., Shaw A.T. (2018). Tumour Heterogeneity and Resistance to Cancer Therapies. Nat. Rev. Clin. Oncol..

[18] Bedard P.L., Hansen A.R., Ratain M.J., Siu L.L. (2013). Tumour Heterogeneity in the Clinic. Nature.

[19] Kumar R., Godavarthy P.S., Krause D.S. (2018). The Bone Marrow Microenvironment in Health and Disease at a Glance. J. Cell Sci..

[20] Basak G., Srivastava A., Malhotra R., Carrier E. (2009). Multiple Myeloma Bone Marrow Niche. Curr. Pharm. Biotechnol..

[21] Lopes R., Caetano J., Ferreira B., Barahona F., Carneiro E.A., João C. (2021). The Immune Microenvironment in Multiple Myeloma: Friend or Foe?. Cancers.

[22] Kawano Y., Moschetta M., Manier S., Glavey S., Görgün G.T., Roccaro A.M., Anderson K.C., Ghobrial I.M. (2015). Targeting the Bone Marrow Microenvironment in Multiple Myeloma. Immunol. Rev..

[23] Zhang L., Tai Y.T., Ho M., Xing L., Chauhan D., Gang A., Qiu L., Anderson K.C. (2017). Regulatory B Cell-Myeloma Cell Interaction Confers Immunosuppression and Promotes Their Survival in the Bone Marrow Milieu. Blood Cancer J..

[24] Paiva B., Azpilikueta A., Puig N., Ocio E.M., Sharma R., Oyajobi B.O., Labiano S., San-Segundo L., Rodriguez A., Aires-Mejia I. (2015). PD-L1/PD-1 Presence in the Tumor Microenvironment and Activity of PD-1 Blockade in Multiple Myeloma. Leukemia.

[25] Ray A., Das D.S., Song Y., Richardson P., Munshi N.C., Chauhan D., Anderson K.C. (2015). Targeting PD1-PDL1 Immune Checkpoint in Plasmacytoid Dendritic Cell Interactions with T Cells, Natural Killer Cells and Multiple Myeloma Cells. Leukemia.

[26] Wang H., Hu W.M., Xia Z.J., Liang Y., Lu Y., Lin S.X., Tang H. (2019). High Numbers of CD163+ Tumor-Associated Macrophages Correlate with Poor Prognosis in Multiple Myeloma Patients Receiving Bortezomib-Based Regimens. J. Cancer.

[27] Xu R., Li Y., Yan H., Zhang E., Huang X., Chen Q., Chen J., Qu J., Liu Y., He J. (2019). CCL2 Promotes Macrophages-Associated Chemoresistance via MCPIP1 Dual Catalytic Activities in Multiple Myeloma. Cell Death Dis..

[28] Kumar S.K., Rajkumar S.V., Dispenzieri A., Lacy M.Q., Hayman S.R., Buadi F.K., Zeldenrust S.R., Dingli D., Russell S.J., Lust J.A. (2008). Improved Survival in Multiple Myeloma and the Impact of Novel Therapies. Blood.

[29] Lopes R., Ferreira B.V., Caetano J., Barahona F., Carneiro E.A., João C. (2021). Boosting Immunity against Multiple Myeloma. Cancers.

[30] Neri P., Bahlis N.J., Lonial S. (2016). New Strategies in Multiple Myeloma: Immunotherapy as a Novel Approach to Treat Patients with Multiple Myeloma. Clin. Cancer Res..

[31] Charliński G., Vesole D.H., Jurczyszyn A. (2021). Rapid Progress in the Use of Immunomodulatory Drugs and Cereblon E3 Ligase Modulators in the Treatment of Multiple Myeloma. Cancers.

[32] Giuliani N., Ferretti M., Bolzoni M., Storti P., Lazzaretti M., Dalla Palma B., Bonomini S., Martella E., Agnelli L., Neri A. (2012). Increased Osteocyte Death in Multiple Myeloma Patients: Role in Myeloma-Induced Osteoclast Formation. Leukemia.

[33] Delgado-Calle J., Anderson J., Cregor M.D., Hiasa M., Chirgwin J.M., Carlesso N., Yoneda T., Mohammad K.S., Plotkin L.I., Roodman G.D. (2016). Bidirectional Notch Signaling and Osteocyte-Derived Factors in the Bone Marrow Microenvironment Promote Tumor Cell Proliferation and Bone Destruction in Multiple Myeloma. Cancer Res..

[34] Terpos E., Ntanasis-Stathopoulos I., Gavriatopoulou M., Dimopoulos M.A. (2018). Pathogenesis of Bone Disease in Multiple Myeloma: From Bench to Bedside. Blood Cancer J..

[35] Giuliani N., Morandi F., Tagliaferri S., Lazzaretti M., Bonomini S., Crugnola M., Mancini C., Martella E., Ferrari L., Tabilio A. (2007). The Proteasome Inhibitor Bortezomib Affects Osteoblast Differentiation in Vitro and in Vivo in Multiple Myeloma Patients. Blood.

[36] Toscani D., Palumbo C., Dalla Palma B., Ferretti M., Bolzoni M., Marchica V., Sena P., Martella E., Mancini C., Ferri V. (2016). The Proteasome Inhibitor Bortezomib Maintains Osteocyte Viability in Multiple Myeloma Patients by Reducing Both Apoptosis and Autophagy: A New Function for Proteasome Inhibitors. J. Bone Miner. Res..

[37] Hurchla M.A., Garcia-Gomez A., Hornick M.C., Ocio E.M., Li A., Blanco J.F., Collins L., Kirk C.J., Piwnica-Worms D., Vij R. (2013). The Epoxyketone-Based Proteasome Inhibitors Carfilzomib and Orally Bioavailable Oprozomib Have Anti-Resorptive and Bone-Anabolic Activity in Addition to Anti-Myeloma Effects. Leukemia.

[38] Bolomsky A., Schreder M., Meißner T., Hose D., Ludwig H., Pfeifer S., Zojer N. (2014). Immunomodulatory Drugs Thalidomide and Lenalidomide Affect Osteoblast Differentiation of Human Bone Marrow Stromal Cells Invitro. Exp. Hematol..

[39] Terpos E., Dimopoulos M.A., Sezer O. (2007). The Effect of Novel Anti-Myeloma Agents on Bone Metabolism of Patients with Multiple Myeloma. Leukemia.

[40] Munemasa S., Sakai A., Kuroda Y., Okikawa Y., Katayama Y., Asaoku H., Kubo T., Shimose S., Kimura A. (2008). Osteoprogenitor Differentiation Is Not Affected by Immunomodulatory Thalidomide Analogs but Is Promoted by Low Bortezomib Concentration, While Both Agents Suppress Osteoclast Differentiation. Int. J. Oncol..

[41] Anderson G., Gries M., Kurihara N., Honjo T., Anderson J., Donnenberg V., Donnenberg A., Ghobrial I., Mapara M.Y., Stirling D. (2006). Thalidomide Derivative CC-4047 Inhibits Osteoclast Formation by down-Regulation of PU.1. Blood.

[42] Sekiguchi Y., Ichikawa K., Wakabayashi M., Sugimoto K., Tomita S., Izumi H., Nakamura N., Sawada T., Ohta Y., Komatsu N. (2015). Bone Formation Following Lenalidomide-Dexamethasone Combination Therapy in Cases of Multiple Myeloma Refractory to High-Dose Chemotherapy with Bortezomib and Autologous Peripheral Blood Stem Cell Transplantation: Report of a Case and Review of the Literat. Int. J. Clin. Exp. Pathol..

[43] Terpos E., Christoulas D., Kastritis E., Roussou M., Gavriatopoulou M., Migkou M., Gkotzamanidou M., Iakovaki M., Papatheodorou A., Dimopoulos M.A. (2009). The Addition of Bortezomib to the Combination of Lenalidomide and Dexamethasone Increases Bone Formation in Relapsed/Refractory Myeloma: A Prospective Study in 91 Patients. Blood.

[44] Tsuda H., Yamasaki H., Tsuji T., Yokoo E. (2012). Therapy with Lenalidomide plus Dexamethasone-Induced Bone Formation in a Patient with Refractory Multiple Myeloma. Int. J. Hematol..

[45] Terpos E., Zamagni E., Lentzsch S., Drake M.T., García-Sanz R., Abildgaard N., Ntanasis-Stathopoulos I., Schjesvold F., de la Rubia J., Kyriakou C. (2021). Treatment of Multiple Myeloma-Related Bone Disease: Recommendations from the Bone Working Group of the International Myeloma Working Group. Lancet Oncol..

[46] Storti P., Bolzoni M., Donofrio G., Airoldi I., Guasco D., Toscani D., Martella E., Lazzaretti M., Mancini C., Agnelli L. (2013). Hypoxia-Inducible Factor (HIF)-1α Suppression in Myeloma Cells Blocks Tumoral Growth in Vivo Inhibiting Angiogenesis and Bone Destruction. Leukemia.

[47] Calcinotto A., Ponzoni M., Ria R., Grioni M., Cattaneo E., Villa I., Bertilaccio M.T.S., Chesi M., Rubinacci A., Tonon G. (2015). Modifications of the Mouse Bone Marrow Microenvironment Favor Angiogenesis and Correlate with Disease Progression from Asymptomatic to Symptomatic Multiple Myeloma. Oncoimmunology.

[48] Vacca A., Ribatti D. (2006). Bone Marrow Angiogenesis in Multiple Myeloma. Leukemia.

[49] Scavelli C., Di Pietro G., Cirulli T., Coluccia M., Boccarelli A., Giannini T., Mangialardi G., Bertieri R., Coluccia A.M.L., Ribatti D. (2007). Zoledronic Acid Affects Over-Angiogenic Phenotype of Endothelial Cells in Patients with Multiple Myeloma. Mol. Cancer Ther..

[50] Roccaro A.M., Hideshima T., Raje N., Kumar S., Ishitsuka K., Yasui H., Shiraishi N., Ribatti D., Nico B., Vacca A. (2006). Bortezomib Mediates Antiangiogenesis in Multiple Myeloma via Direct and Indirect Effects on Endothelial Cells. Cancer Res..

[51] Chanan-Khan A.A., Swaika A., Paulus A., Kumar S.K., Mikhael J.R., Rajkumar S.V., Dispenzieri A., Lacy M.Q. (2013). Pomalidomide: The New Immunomodulatory Agent for the Treatment of Multiple Myeloma. Blood Cancer J..

[52] Lu L., Payvandi F., Wu L., Zhang L.H., Hariri R.J., Man H.W., Chen R.S., Muller G.W., Hughes C.C.W., Stirling D.I. (2009). The Anti-Cancer Drug Lenalidomide Inhibits Angiogenesis and Metastasis via Multiple Inhibitory Effects on Endothelial Cell Function in Normoxic and Hypoxic Conditions. Microvasc. Res..

[53] Vacca A., Scavelli C., Montefusco V., Di Pietro G., Neri A., Mattioli M., Bicciato S., Nico B., Ribatti D., Dammacco F. (2005). Thalidomide Downregulates Angiogenic Genes in Bone Marrow Endothelial Cells of Patients with Active Multiple Myeloma. J. Clin. Oncol..

[54] Ullah T.R. (2019). The Role of CXCR4 in Multiple Myeloma: Cells’ Journey from Bone Marrow to beyond. J. Bone Oncol..

[55] Azab A.K., Runnels J.M., Pitsillides C., Moreau A.S., Azab F., Leleu X., Jia X., Wright R., Ospina B., Carlson A.L. (2009). CXCR4 Inhibitor AMD3100 Disrupts the Interaction of Multiple Myeloma Cells with the Bone Marrow Microenvironment and Enhances Their Sensitivity to Therapy. Blood.

[56] Caers J., Deleu S., Belaid Z., De Raeve H., Van Valckenborgh E., De Bruyne E., DeFresne M.P., Van Riet I., Van Camp B., Vanderkerken K. (2007). Neighboring Adipocytes Participate in the Bone Marrow Microenvironment of Multiple Myeloma Cells. Leukemia.

[57] Liu Z., Xu J., He J., Liu H., Lin P., Wan X., Navone N.M., Tong Q., Kwak L.W., Orlowski R.Z. (2015). Mature Adipocytes in Bone Marrow Protect Myeloma Cells against Chemotherapy through Autophagy Activation. Oncotarget.

[58] Shu L., Li J., Chen S., Huang H.-Y., Li Y., Liang Y. (2021). Bone Marrow Adipocyte Shapes Metabolism and Immunity in Tumor Microenvironment to Promote Multiple Myeloma. Blood,.

[59] Bahlis N.J. (2012). Darwinian Evolution and Tiding Clones in Multiple Myeloma. Blood.

[60] Peled J.U., Devlin S.M., Staffas A., Lumish M., Khanin R., Littmann E.R., Ling L., Kosuri S., Maloy M., Slingerland J.B. (2017). Intestinal Microbiota and Relapse after Hematopoietic-Cell Transplantation. J. Clin. Oncol..

[61] Pianko M.J., Devlin S.M., Littmann E.R., Chansakul A., Mastey D., Salcedo M., Fontana E., Ling L., Tavitian E., Slingerland J.B. (2019). Minimal Residual Disease Negativity in Multiple Myeloma Is Associated with Intestinal Microbiota Composition. Blood Adv..

[62] McCachren S.S., Dhodapkar K.M., Dhodapkar M.V. (2021). Co-Evolution of Immune Response in Multiple Myeloma: Implications for Immune Prevention. Front. Immunol..

[63] Silva A., Silva M.C., Sudalagunta P., DIstler A., Jacobson T., Collins A., Nguyen T., Song J., Chen D.T., Chen L. (2017). An Ex Vivo Platform for the Prediction of Clinical Response in Multiple Myeloma. Cancer Res..

[64] Sudalagunta P., Silva M.C., Canevarolo R.R., Alugubelli R.R., DeAvila G., Tungesvik A., Perez L., Gatenby R., Gillies R., Baz R. (2020). A Pharmacodynamic Model of Clinical Synergy in Multiple Myeloma. EBioMedicine.

[65] Masters J.R. (2002). HeLa Cells 50 Years on: The Good, the Bad and the Ugly. Nat. Rev. Cancer.

[66] Raulf M. (2020). T cell: Primary culture from peripheral blood. Methods in Molecular Biology.

[67] Dronkers E., Moerkamp A.T., van Herwaarden T., Goumans M.J., Smits A.M. (2018). The Isolation and Culture of Primary Epicardial Cells Derived from Human Adult and Fetal Heart Specimens. J. Vis. Exp..

[68] Fitzgerald A.A., Li E., Weiner L.M. (2020). 3D Culture Systems for Exploring Cancer Immunology. Cancers.

[69] Barbosa M.A.G., Xavier C.P.R., Pereira R.F., Petrikaitė V., Vasconcelos M.H. (2021). 3D Cell Culture Models as Recapitulators of the Tumor Microenvironment for the Screening of Anti-Cancer Drugs. Cancers.

[70] Knight E., Przyborski S. (2015). Advances in 3D Cell Culture Technologies Enabling Tissue-like Structures to Be Created in Vitro. J. Anat..

[71] Fuchs E., Tumbar T., Guasch G. (2004). Socializing with the Neighbors: Stem Cells and Their Niche. Cell.

[72] Petersen O.W., Ronnov-Jessen L., Howlett A.R., Bissell M.J. (1992). Interaction with Basement Membrane Serves to Rapidly Distinguish Growth and Differentiation Pattern of Normal and Malignant Human Breast Epithelial Cells. Proc. Natl. Acad. Sci. USA.

[73] Sarin V., Yu K., Ferguson I.D., Gugliemini O., Nix M.A., Hann B., Sirota M., Wiita A.P. (2020). Evaluating the Efficacy of Multiple Myeloma Cell Lines as Models for Patient Tumors via Transcriptomic Correlation Analysis. Leukemia.

[74] Santo V.E., Rebelo S.P., Estrada M.F., Alves P.M., Boghaert E., Brito C. (2017). Drug Screening in 3D in Vitro Tumor Models: Overcoming Current Pitfalls of Efficacy Read-Outs. Biotechnol. J..

[75] Maiso P., Huynh D., Moschetta M., Sacco A., Aljawai Y., Mishima Y., Asara J.M., Roccaro A.M., Kimmelman A.C., Ghobrial I.M. (2015). Metabolic Signature Identifies Novel Targets for Drug Resistance in Multiple Myeloma. Cancer Res..

[76] Gavriatopoulou M., Paschou S.A., Ntanasis-stathopoulos I., Dimopoulos M.A. (2021). Metabolic Disorders in Multiple Myeloma. Int. J. Mol. Sci..

[77] Baker B.M., Chen C.S. (2012). Deconstructing the Third Dimension-How 3D Culture Microenvironments Alter Cellular Cues. J. Cell Sci..

[78] Kleinman H.K., Philp D., Hoffman M.P. (2003). Role of the Extracellular Matrix in Morphogenesis. Curr. Opin. Biotechnol..

[79] Riedl A., Schlederer M., Pudelko K., Stadler M., Walter S., Unterleuthner D., Unger C., Kramer N., Hengstschläger M., Kenner L. (2017). Comparison of Cancer Cells in 2D vs 3D Culture Reveals Differences in AKT-MTOR-S6K Signaling and Drug Responses. J. Cell Sci..

[80] Radl J., Croese J.W., Zurcher C., Van Den Enden-Vieveen M.H.M., De Leeuw A.M. (1988). Animal Model of Human Disease. Multiple Myeloma. Am. J. Pathol..

[81] Yaccoby S., Barlogie B., Epstein J. (1998). Primary Myeloma Cells Growing in SCID-Hu Mice: A Model for Studying the Biology and Treatment of Myeloma and Its Manifestations. Blood.

[82] Mestas J., Hughes C.C.W. (2004). Of Mice and Not Men: Differences between Mouse and Human Immunology. J. Immunol..

[83] Ben-David U., Ha G., Tseng Y.Y., Greenwald N.F., Oh C., Shih J., McFarland J.M., Wong B., Boehm J.S., Beroukhim R. (2017). Patient-Derived Xenografts Undergo Mouse-Specific Tumor Evolution. Nat. Genet..

[84] Meyer L.H., Debatin K.M. (2011). Diversity of Human Leukemia Xenograft Mouse Models: Implications for Disease Biology. Cancer Res..

[85] Hanna T.P., King W.D., Thibodeau S., Jalink M., Paulin G.A., Harvey-Jones E., O’Sullivan D.E., Booth C.M., Sullivan R., Aggarwal A. (2020). Mortality Due to Cancer Treatment Delay: Systematic Review and Meta-Analysis. BMJ.

[86] Cheon D.J., Orsulic S. (2011). Mouse Models of Cancer. Annu. Rev. Pathol..

[87] Workman P., Aboagye E.O., Balkwill F., Balmain A., Bruder G., Chaplin D.J., Double J.A., Everitt J., Farningham D.A.H., Glennie M.J. (2010). Guidelines for the Welfare and Use of Animals in Cancer Research. Br. J. Cancer.

[88] Carrel A. (1912). On the Permanent Life of Tissues Outside of the Organism. J. Exp. Med..

[89] Hamburger A.W., Salmon S.E. (1977). Primary Bioassay of Human Tumor Stem Cells. Science.

[90] Magdeldin T., López-Dávila V., Pape J., Cameron G.W.W., Emberton M., Loizidou M., Cheema U. (2017). Engineering a Vascularised 3D in Vitro Model of Cancer Progression. Sci. Rep..

[91] Wang X., Sun Q., Pei J. (2018). Microfluidic-Based 3D Engineered Microvascular Networks and Their Applications in Vascularized Microtumor Models. Micromachines.

[92] Cheema U., Brown R.A., Alp B., MacRobert A.J. (2008). Spatially Defined Oxygen Gradients and Vascular Endothelial Growth Factor Expression in an Engineered 3D Cell Model. Cell. Mol. Life Sci..

[93] Driehuis E., Kretzschmar K., Clevers H. (2020). Establishment of Patient-Derived Cancer Organoids for Drug-Screening Applications. Nat. Protoc..

[94] Zdzisińska B., Roliński J., Piersiak T., Kandefer-Szerszeń M. (2009). A Comparison of Cytokine Production in 2-Dimensional and 3-Dimensional Cultures of Bone Marrow Stromal Cells of Muliple Myeloma Patients in Response to RPMI8226 Myeloma Cells. Folia Histochem. Cytobiol..

[95] De la Puente P., Muz B., Gilson R.C., Azab F., Luderer M., King J., Achilefu S., Vij R., Azab A.K. (2015). 3D Tissue-Engineered Bone Marrow as a Novel Model to Study Pathophysiology and Drug Resistance in Multiple Myeloma. Biomaterials.

[96] Spelat R., Ferro F., Contessotto P., Warren N.J., Marsico G., Armes S.P., Pandit A. (2020). A Worm Gel-Based 3D Model to Elucidate the Paracrine Interaction between Multiple Myeloma and Mesenchymal Stem Cells. Mater. Today Bio..

[97] Kirshner J., Thulien K.J., Martin L.D., Marun C.D., Reiman T., Belch A.R., Pilarski L.M. (2008). A Unique Three-Dimensional Model for Evaluating the Impact of Therapy on Multiple Myeloma. Blood.

[98] Matsui W., Wang Q., Barber J.P., Brennan S., Smith B.D., Borrello I., McNiece I., Lin L., Ambinder R.F., Peacock C. (2008). Clonogenic Multiple Myeloma Progenitors, Stem Cell Properties, and Drug Resistance. Cancer Res..

[99] Zhang G., Miao F., Xu J., Wang R. (2020). Mesenchymal Stem Cells from Bone Marrow Regulate Invasion and Drug Resistance of Multiple Myeloma Cells by Secreting Chemokine CXCL13. Bosn. J. Basic Med. Sci..

[100] Parikh M.R., Belch A.R., Pilarski L.M., Kirshner J. (2014). A Three-Dimensional Tissue Culture Model to Study Primary Human Bone Marrow and Its Malignancies. J. Vis. Exp..

[101] Kirshner J., Kirshnan A., Nathwani N., Htut M., Rosenzweig M., Karanes C., Firoozeh S., Rosen S. (2020). Reconstructed bone (r-bone): A patient-derived 3D culture platform for prediction of clinical outcomes in multiple myeloma. Proceedings of the Annual Meeting of the American Association for Cancer Research.

[102] Huang Y.H., Molavi O., Alshareef A., Haque M., Wang Q., Chu M.P., Venner C.P., Sandhu I., Peters A.C., Lavasanifar A. (2018). Constitutive Activation of STAT3 in Myeloma Cells Cultured in a Three-Dimensional, Reconstructed Bone Marrow Model. Cancers.

[103] Huang Y.H., Almowaled M., Li J., Venner C., Sandhu I., Peters A., Lavasanifar A., Lai R. (2021). Three-Dimensional Reconstructed Bone Marrow Matrix Culture Improves the Viability of Primary Myeloma Cells in-Vitro via a Stat3-Dependent Mechanism. Curr. Issues Mol. Biol..

[104] Musolino C., Allegra A., Innao V., Allegra A.G., Pioggia G., Gangemi S. (2017). Inflammatory and Anti-Inflammatory Equilibrium, Proliferative and Antiproliferative Balance: The Role of Cytokines in Multiple Myeloma. Mediat. Inflamm..

[105] Caillot M., Zylbersztejn F., Maitre E., Bourgeais J., Hérault O., Sola B. (2020). ROS Overproduction Sensitises Myeloma Cells to Bortezomib-Induced Apoptosis and Alleviates Tumour Microenvironment-Mediated Cell Resistance. Cells.

[106] Lipchick B.C., Fink E.E., Nikiforov M.A. (2016). Oxidative Stress and Proteasome Inhibitors in Multiple Myeloma. Pharmacol. Res..

[107] Perez C., Botta C., Zabaleta A., Puig N., Cedena M.T., Goicoechea I., Alameda D., José-Eneriz E.S., Merino J., Rodríguez-Otero P. (2020). Immunogenomic Identification and Characterization of Granulocytic Myeloid-Derived Suppressor Cells in Multiple Myeloma. Blood.

[108] Yu S., Ren X., Li L. (2022). Myeloid-Derived Suppressor Cells in Hematologic Malignancies: Two Sides of the Same Coin. Exp. Hematol. Oncol..

[109] Cucè M., Gallo Cantafio M.E., Siciliano M.A., Riillo C., Caracciolo D., Scionti F., Staropoli N., Zuccalà V., Maltese L., Di Vito A. (2019). Trabectedin Triggers Direct and NK-Mediated Cytotoxicity in Multiple Myeloma. J. Hematol. Oncol..

[110] Ali J.Y.H., Fitieh A.M., Ismail I.H. (2022). The Role of DNA Repair in Genomic Instability of Multiple Myeloma. Int. J. Mol. Sci..

[111] Hutmacher D.W., Horch R.E., Loessner D., Rizzi S., Sieh S., Reichert J.C., Clements J.A., Beier J.P., Arkudas A., Bleiziffer O. (2009). Translating Tissue Engineering Technology Platforms into Cancer Research. J. Cell. Mol. Med..

[112] Khan A.O., Colombo M., Reyat J.S., Wang G., Rodriguez-Romera A., Wen W.X., Murphy L., Grygielska B., Mahoney C., Stone A. (2022). Human Bone Marrow Organoids for Disease Modelling, Discovery and Validation of Therapeutic Targets in Hematological Malignancies. BioRxiv.

[113] Braham M.V.J., Minnema M.C., Aarts T., Sebestyen Z., Straetemans T., Vyborova A., Kuball J., Öner F.C., Robin C., Alblas J. (2018). Cellular Immunotherapy on Primary Multiple Myeloma Expanded in a 3D Bone Marrow Niche Model. Oncoimmunology.

[114] Braham M.V.J., Deshantri A.K., Minnema M.C., Öner F.C., Schiffelers R.M., Fens M.H.A.M., Alblas J. (2018). Liposomal Drug Delivery in an in Vitro 3D Bone Marrow Model for Multiple Myeloma. Int. J. Nanomedicine.

[115] Van Diest E., López P.H., Meringa A.D., Vyborova A., Karaiskaki F., Heijhuurs S., Bormin J.G., Van Dooremalen S., Nicolasen M.J.T., Gatti L.C.D.E. (2021). Gamma Delta TCR Anti-CD3 Bispecific Molecules (GABs) as Novel Immunotherapeutic Compounds. J. Immunother. Cancer.

[116] Braham M.V.J., Alblas J., Dhert W.J.A., Öner F.C., Minnema M.C. (2019). Possibilities and Limitations of an in Vitro Three-Dimensional Bone Marrow Model for the Prediction of Clinical Responses in Patients with Relapsed Multiple Myeloma. Haematologica.

[117] Braham M.V.J., Ahlfeld T., Akkineni A.R., Minnema M.C., Dhert W.J.A., Öner F.C., Robin C., Lode A., Gelinsky M., Alblas J. (2018). Endosteal and Perivascular Subniches in a 3D Bone Marrow Model for Multiple Myeloma. Tissue Eng. Part C Methods.

[118] Augustine R., Kalva S.N., Ahmad R., Zahid A.A., Hasan S., Nayeem A., McClements L., Hasan A. (2021). 3D Bioprinted Cancer Models: Revolutionizing Personalized Cancer Therapy. Transl. Oncol..

[119] Rodriguez C., Aleman J., Almeida-Porada G., Porada C., Nikiforov M., Skardal A. (2019). High Throughput 3D Bioprinting of Patient-Derived Multiple Myeloma Organoid Models for Niche Recapitulation and Chemosensitivity Assessment. Clin. Lymphoma. Myeloma Leuk..

[120] Wu D., Wang Z., Li J., Song Y., Perez M.E.M., Wang Z., Cao X., Cao C., Maharjan S., Anderson K.C. (2022). A 3D-Bioprinted Multiple Myeloma Model. Adv. Healthc. Mater..

[121] Davoodi E., Sarikhani E., Montazerian H., Ahadian S., Costantini M., Swieszkowski W., Willerth S.M., Walus K., Mofidfar M., Toyserkani E. (2020). Extrusion and Microfluidic-Based Bioprinting to Fabricate Biomimetic Tissues and Organs. Adv. Mater. Technol..

[122] Ma J., Wang Y., Liu J. (2018). Bioprinting of 3D Tissues/Organs Combined with Microfluidics. RSC Adv..

[123] Kawano Y., Kikukawa Y., Fujiwara S., Wada N., Okuno Y., Mitsuya H., Hata H. (2013). Hypoxia Reduces CD138 Expression and Induces an Immature and Stem Cell-like Transcriptional Program in Myeloma Cells. Int. J. Oncol..

[124] Kyle A.H., Huxham L.A., Chiam A.S.J., Sim D.H., Minchinton A.I. (2004). Direct Assessment of Drug Penetration into Tissue Using a Novel Application of Three-Dimensional Cell Culture. Cancer Res..

[125] Muz B., Kusdono H.D., Azab F., de la Puente P., Federico C., Fiala M., Vij R., Salama N.N., Azab A.K. (2017). Tariquidar Sensitizes Multiple Myeloma Cells to Proteasome Inhibitors via Reduction of Hypoxia-Induced P-Gp-Mediated Drug Resistance. Leuk. Lymphoma.

[126] Sun J., Muz B., Alhallak K., Markovic M., Gurley S., Wang Z., Guenthner N., Wasden K., Fiala M., King J. (2020). Targeting CD47 as a Novel Immunotherapy for Multiple Myeloma. Cancers.

[127] Alhallak K., Jeske A., de la Puente P., Sun J., Fiala M., Azab F., Muz B., Sahin I., Vij R., DiPersio J.F. (2021). A Pilot Study of 3D Tissue-Engineered Bone Marrow Culture as a Tool to Predict Patient Response to Therapy in Multiple Myeloma. Sci. Rep..

[128] Jakubikova J., Cholujova D., Hideshima T., Gronesova P., Soltysova A., Harada T., Joo J., Kong S.Y., Szalat R.E., Richardson P.G. (2016). A Novel 3D Mesenchymal Stem Cell Model of the Multiple Myeloma Bone Marrow Niche: Biologic and Clinical Applications. Oncotarget.

[129] Waldschmidt J.M., Fruttiger S.J., Wider D., Jung J., Thomsen A.R., Hartmann T.N., Duyster J., Hug M.J., Azab K.A., Jung M. (2022). Ex Vivo Propagation in a Novel 3D High-Throughput Co-Culture System for Multiple Myeloma. J. Cancer Res. Clin. Oncol..

[130] Gu J., Huang X., Zhang Y., Bao C., Zhou Z., Jin J. (2021). Cytokine Profiles in Patients with Newly Diagnosed Multiple Myeloma: Survival Is Associated with IL-6 and IL-17A Levels. Cytokine.

[131] Müller S.J., Waldschmidt J.M., Senger J., Wider D., Thomsen A.R., Ihorst G., Duyster J., Hug M.J., Jung M., Wäsch R. (2016). Testing Novel Anti-Multiple Myeloma (MM) Agents in a Suitable Three-Dimensional (3D) Co-Culture Platform. Blood.

[132] Müller S.J., Wider D., Thomsen A.R., Follo M., Waldschmidt J.M., Ihorst G., Dold S.M., Felthaus J., Senger J., Schüler J. (2017). Epigenetic Modifications of the Bone Marrow (BM) Niche in Multiple Myeloma (MM)-a Three-Dimensional (3D) in Vitro Approach. Blood.

[133] Gurski L.A., Petrelli N.J., Jia X., Farach-Carson M.C. (2010). 3D Matrices for Anti-Cancer Drug Testing and Development. Oncol. Issues.

[134] Ahn Y.H., Ren L., Kim S.M., Seo S.H., Jung C.R., Kim D.S., Noh J.Y., Lee S.Y., Lee H., Cho M.Y. (2020). A Three-Dimensional Hyaluronic Acid-Based Niche Enhances the Therapeutic Efficacy of Human Natural Killer Cell-Based Cancer Immunotherapy. Biomaterials.

[135] Turtoi M., Anghelache M., Bucatariu S.M., Deleanu M., Voicu G., Safciuc F., Manduteanu I., Fundueanu G., Simionescu M., Calin M. (2021). A Novel Platform for Drug Testing: Biomimetic Three-Dimensional Hyaluronic Acid-Based Scaffold Seeded with Human Hepatocarcinoma Cells. Int. J. Biol. Macromol..

[136] Wang J., Xu W., Qian J., Wang Y., Hou G., Suo A. (2022). Photo-Crosslinked Hyaluronic Acid Hydrogel as a Biomimic Extracellular Matrix to Recapitulate in Vivo Features of Breast Cancer Cells. Colloids Surf. B Biointerfaces.

[137] Xiao W., Ehsanipour A., Sohrabi A., Seidlits S.K. (2018). Hyaluronic-Acid Based Hydrogels for 3-Dimensional Culture of Patient-Derived Glioblastoma Cells. J. Vis. Exp..

[138] Narayanan N.K., Duan B.I.N., Butcher J.T., Mazumder A., Narayanan B.A. (2014). Characterization of Multiple Myeloma Clonal Cell Expansion and Stromal Wnt/β-Catenin Signaling in Hyaluronic Acid-Based 3D Hydrogel. In Vivo.

[139] Leiva O., Leon C., Kah Ng S., Mangin P., Gachet C., Ravid K. (2018). The Role of Extracellular Matrix Stiffness in Megakaryocyte and Platelet Development and Function. Am. J. Hematol..

[140] Ishihara S., Haga H. (2022). Matrix Stiffness Contributes to Cancer Progression by Regulating Transcription Factors. Cancers.

[141] Isomursu A., Park K.Y., Hou J., Cheng B., Mathieu M., Shamsan G.A., Fuller B., Kasim J., Mahmoodi M.M., Lu T.J. (2022). Directed Cell Migration towards Softer Environments. Nat. Mater..

[142] Sidhu I., Barwe S.P., Gopalakrishnapillai A. (2021). The Extracellular Matrix: A Key Player in the Pathogenesis of Hematologic Malignancies. Blood Rev..

[143] Ferrarini M., Steimberg N., Ponzoni M., Belloni D., Berenzi A., Girlanda S., Caligaris-Cappio F., Mazzoleni G., Ferrero E. (2013). Ex-Vivo Dynamic 3-D Culture of Human Tissues in the RCCS^TM^ Bioreactor Allows the Study of Multiple Myeloma Biology and Response to Therapy. PLoS ONE.

[144] Ferrarini M., Steimberg N., Boniotti J., Berenzi A., Belloni D., Mazzoleni G., Ferrero E. (2017). 3D-dynamic culture models of multiple myeloma. Methods in Molecular Biology.

[145] Belloni D., Heltai S., Ponzoni M., Villa A., Vergani B., Pecciarini L., Marcatti M., Girlanda S., Tonon G., Ciceri F. (2018). Modeling Multiple Myeloma-Bone Marrow Interactions and Response to Drugs in a 3D Surrogate Microenvironment. Haematologica.

[146] Mitteregger R., Vogt G., Rossmanith E., Falkenhagen D. (2000). Rotary Cell Culture System (RCCS): A New Method for Cultivating Hepatocytes on Microcarriers. Int. J. Artif. Organs.

[147] Edmondson R., Broglie J.J., Adcock A.F., Yang L. (2014). Three-Dimensional Cell Culture Systems and Their Applications in Drug Discovery and Cell-Based Biosensors. Assay Drug Dev. Technol..

[148] Gu Y., Zhang W., Sun Q., Hao Y., Zilberberg J., Lee W.Y. (2015). Microbead-Guided Reconstruction of the 3D Osteocyte Network during Microfluidic Perfusion Culture. J. Mater. Chem. B.

[149] Kwapiszewska K., Michalczuk A., Rybka M., Kwapiszewski R., Brzózka Z. (2014). A Microfluidic-Based Platform for Tumour Spheroid Culture, Monitoring and Drug Screening. Lab Chip.

[150] Li X., Valadez A.V., Zuo P., Nie Z. (2012). Microfluidic 3D Cell Culture: Potential Application for Tissue-Based Bioassays. Bioanalysis.

[151] Zhang W., Lee W.Y., Siegel D.S., Tolias P., Zilberberg J. (2014). Patient-Specific 3D Microfluidic Tissue Model for Multiple Myeloma. Tissue Eng. Part C Methods.

[152] Zhang W., Gu Y., Sun Q., Siegel D.S., Tolias P., Yang Z., Lee W.Y., Zilberberg J. (2015). Ex Vivo Maintenance of Primary Human Multiple Myeloma Cells through the Optimization of the Osteoblastic Niche. PLoS ONE.

[153] Zhang W., Gu Y., Hao Y., Sun Q., Konior K., Wang H., Zilberberg J., Lee W.Y. (2015). Well Plate-Based Perfusion Culture Device for Tissue and Tumor Microenvironment Replication. Lab Chip.

[154] Reagan M.R., Mishima Y., Glavey S.V., Zhang Y., Manier S., Lu Z.N., Memarzadeh M., Zhang Y., Sacco A., Aljawai Y. (2014). Investigating Osteogenic Differentiation in Multiple Myeloma Using a Novel 3D Bone Marrow Niche Model. Blood.

[155] Xu S., Santini G.C., De Veirman K., Broek I.V., Leleu X., De Becker A., Van Camp B., Vanderkerken K., Van Riet I. (2013). Upregulation of MiR-135b Is Involved in the Impaired Osteogenic Differentiation of Mesenchymal Stem Cells Derived from Multiple Myeloma Patients. PLoS ONE.

[156] Fairfield H., Falank C., Farrell M., Vary C., Boucher J.M., Driscoll H., Liaw L., Rosen C.J., Reagan M.R. (2019). Development of a 3D Bone Marrow Adipose Tissue Model. Bone.

[157] Trotter T.N., Gibson J.T., Sherpa T.L., Gowda P.S., Peker D., Yang Y. (2016). Adipocyte-Lineage Cells Support Growth and Dissemination of Multiple Myeloma in Bone. Am. J. Pathol..

[158] Watanabe Y., Dahlman E.L., Leder K.Z., Hui S.K. (2016). A Mathematical Model of Tumor Growth and Its Response to Single Irradiation. Theor. Biol. Med. Model..

[159] Laleh N.G., Loeffler C.M.L., Grajek J., Staňková K., Pearson A.T., Muti H.S., Trautwein C., Enderling H., Poleszczuk J., Kather J.N. (2022). Classical Mathematical Models for Prediction of Response to Chemotherapy and Immunotherapy. PLoS Comput. Biol..

[160] Khin Z.P., Ribeiro M.L.C., Jacobson T., Hazlehurst L., Perez L., Baz R., Shain K., Silva A.S. (2014). A Preclinical Assay for Chemosensitivity in Multiple Myeloma. Cancer Res..

[161] Silva A., Jacobson T., Meads M., Distler A., Shain K. (2015). An Organotypic High Throughput System for Characterization of Drug Sensitivity of Primary Multiple Myeloma Cells. J. Vis. Exp..

[162] Akaike H. (1974). Akaike, H. A new look at the statistical model identification. Selected Papers of Hirotugu Akaike.

[163] Rajkumar S.V. (2022). Multiple Myeloma: 2022 Update on Diagnosis, Risk Stratification, and Management. Am. J. Hematol..

[164] Guang M.H.Z., McCann A., Bianchi G., Zhang L., Dowling P., Bazou D., O’Gorman P., Anderson K.C. (2018). Overcoming Multiple Myeloma Drug Resistance in the Era of Cancer ‘Omics. ’ Leuk. Lymphoma.

[165] Kapałczyńska M., Kolenda T., Przybyła W., Zajączkowska M., Teresiak A., Filas V., Ibbs M., Bliźniak R., Łuczewski Ł., Lamperska K. (2018). 2D and 3D Cell Cultures—A Comparison of Different Types of Cancer Cell Cultures. Arch. Med. Sci..

[166] Votanopoulos K.I., Forsythe S., Sivakumar H., Mazzocchi A., Aleman J., Miller L., Levine E., Triozzi P., Skardal A. (2019). Model of Patient-Specific Immune-Enhanced Organoids for Immunotherapy Screening: Feasibility Study. Ann. Surg. Oncol..

[167] Rebelo S.P., Pinto C., Martins T.R., Harrer N., Estrada M.F., Loza-Alvarez P., Cabeçadas J., Alves P.M., Gualda E.J., Sommergruber W. (2018). 3D-3-Culture: A Tool to Unveil Macrophage Plasticity in the Tumour Microenvironment. Biomaterials.

[168] Courau T., Bonnereau J., Chicoteau J., Bottois H., Remark R., Assante Miranda L., Toubert A., Blery M., Aparicio T., Allez M. (2019). Cocultures of Human Colorectal Tumor Spheroids with Immune Cells Reveal the Therapeutic Potential of MICA/B and NKG2A Targeting for Cancer Treatment. J. Immunother. Cancer.

[169] Korman A.J., Peggs K.S., Allison J.P. (2006). Checkpoint Blockade in Cancer Immunotherapy. Adv. Immunol..

[170] Boucherit N., Gorvel L., Olive D. (2020). 3D Tumor Models and Their Use for the Testing of Immunotherapies. Front. Immunol..

[171] Benton G., Kleinman H.K., George J., Arnaoutova I. (2011). Multiple Uses of Basement Membrane-like Matrix (BME/Matrigel) in Vitro and in Vivo with Cancer Cells. Int. J. Cancer,.

[172] Wolf K., Alexander S., Schacht V., Coussens L.M., von Andrian U.H., van Rheenen J., Deryugina E., Friedl P. (2009). Collagen-Based Cell Migration Models in Vitro and in Vivo. Semin. Cell Dev. Biol..

[173] Goodarzi K., Rao S.S. (2021). Hyaluronic Acid-Based Hydrogels to Study Cancer Cell Behaviors. J. Mater. Chem. B.

[174] Rosso F., Giordano A., Barbarisi M., Barbarisi A. (2004). From Cell-ECM Interactions to Tissue Engineering. J. Cell. Physiol..

[175] Kaur S., Kaur I., Rawal P., Tripathi D.M., Vasudevan A. (2021). Non-Matrigel Scaffolds for Organoid Cultures. Cancer Lett..

[176] Breslin S., O’Driscoll L. (2013). Three-Dimensional Cell Culture: The Missing Link in Drug Discovery. Drug Discov. Today.

[177] Kleinman H.K., Martin G.R. (2005). Matrigel: Basement Membrane Matrix with Biological Activity. Semin. Cancer Biol..

[178] Fallacara A., Baldini E., Manfredini S., Vertuani S. (2018). Hyaluronic Acid in the Third Millennium. Polymers.

[179] Huang J. (2019). 3D Cell Culture On VitroGel System. J. Cytol. Tissue Biol..

[180] Toivonen S., Malinen M.M., Küblbeck J., Petsalo A., Urtti A., Honkakoski P., Otonkoski T. (2016). Regulation of Human Pluripotent Stem Cell-Derived Hepatic Cell Phenotype by Three-Dimensional Hydrogel Models. Tissue Eng. Part A.

[181] Graf B.W., Boppart S.A. (2010). Imaging and Analysis of Three-Dimensional Cell Culture Models. Methods Mol. Biol.,.

[182] Carter E.P., Roozitalab R., Gibson S.V., Grose R.P. (2021). Tumour Microenvironment 3D-Modelling: Simplicity to Complexity and Back Again. Trends Cancer.

[183] Corning® Matrigel® Matrix Frequently Asked Questions. https://www.corning.com/catalog/cls/documents/faqs/CLS-DL-CC-026.pdf.

[184] Pinto M.P., Jacobsen B.M., Horwitz K.B. (2011). An Immunohistochemical Method to Study Breast Cancer Cell Subpopulations and Their Growth Regulation by Hormones in Three-Dimensional Cultures. Front. Endocrinol..

[185] Benien P., Swami A. (2014). 3D Tumor Models: History, Advances and Future Perspectives. Future Oncol..

[186] Pulak R. (2006). Techniques for Analysis, Sorting, and Dispensing of C. Elegans on the COPAS Flow-Sorting System. Methods Mol. Biol..

[187] Paulus A., Sharma M., Paulus S.M., Menghani R., Rodriguez E.M., Frank R.D., Cooper G., Hodge D., Roy V., Fonseca R. (2016). Genomic Variability in Multiple Myeloma (MM) Patients By Race: An Analysis of the Publically Available Mmrf Commpass Study Database. Blood.

[188] Awada H., Thapa B., Awada H., Dong J., Gurnari C., Hari P., Dhakal B. (2021). A Comprehensive Review of the Genomics of Multiple Myeloma: Evolutionary Trajectories, Gene Expression Profiling, and Emerging Therapeutics. Cells.

[189] Catenacci D.V.T. (2015). Next-Generation Clinical Trials: Novel Strategies to Address Thechallenge of Tumor Molecular Heterogeneity. Mol. Oncol..

[190] Lee N., Moon S.Y., Lee J.H., Park H.K., Kong S.Y., Bang S.M., Lee J.H., Yoon S.S., Lee D.S. (2017). Discrepancies between the Percentage of Plasma Cells in Bone Marrow Aspiration and BM Biopsy: Impact on the Revised IMWG Diagnostic Criteria of Multiple Myeloma. Blood Cancer J..

[191] Serras A.S., Rodrigues J.S., Cipriano M., Rodrigues A.V., Oliveira N.G., Miranda J.P. (2021). A Critical Perspective on 3D Liver Models for Drug Metabolism and Toxicology Studies. Front. Cell Dev. Biol..

[192] Bajaj P., Chowdhury S.K., Yucha R., Kelly E.J., Xiao G. (2018). Emerging Kidney Models to Investigate Metabolism, Transport, and Toxicity of Drugs and Xenobiotics. Drug Metab. Dispos..

[193] Skardal A., Murphy S.V., Devarasetty M., Mead I., Kang H.W., Seol Y.J., Zhang Y.S., Shin S.R., Zhao L., Aleman J. (2017). Multi-Tissue Interactions in an Integrated Three-Tissue Organ-on-a-Chip Platform. Sci. Rep..

[194] Skardal A., Aleman J., Forsythe S., Rajan S., Murphy S., Devarasetty M., Pourhabibi Zarandi N., Nzou G., Wicks R., Sadri-Ardekani H. (2020). Drug Compound Screening in Single and Integrated Multi-Organoid Body-on-a-Chip Systems. Biofabrication.

